# Supplementary material for: Mechanisms of axoneme and centriole elimination in Naegleria gruberi
Source: EMBO Rep. 2024 Dec 2;26(2):385–406. doi: 10.1038/s44319-024-00329-w (PMC11772885; doi:10.1038/s44319-024-00329-w)
Supplement: Supplementary file 2 — Movie EV1 [file 44319_2024_329_MOESM2_ESM.zip › Movie EV1/Movie EV1.rtf]

Movie EV1DIC movie of flagellated cell (~t=100 min after transformation onset). Reversal was induced by pressure of the coverslip. Time in sec. Scalebar: 5 µm.
