# Supplementary material for: Mechanisms of axoneme and centriole elimination in Naegleria gruberi
Source: EMBO Rep. 2024 Dec 2;26(2):385–406. doi: 10.1038/s44319-024-00329-w (PMC11772885; doi:10.1038/s44319-024-00329-w)
Supplement: Supplementary file 3 — Movie EV2 [file 44319_2024_329_MOESM3_ESM.zip › Movie EV2/Movie EV2.rtf]

Movies EV2Partial Z-projection of a 3D stack of widefield microscopy time-lapse at indicated times after transformation onset. Amoeboid cells immobilized with 10 µM latrunculin at the beginning of the movie, when LysoTracker (green) was added as well; the cabazitaxel-derived microtubule probe SPY650 (magenta) was present since transformation onset. Time in min. Scalebar: 10 µm.
