# Supplementary figures and images for: Mechanisms of axoneme and centriole elimination in Naegleria gruberi
Source: EMBO Rep. 2024 Dec 2;26(2):385–406. doi: 10.1038/s44319-024-00329-w (PMC11772885; doi:10.1038/s44319-024-00329-w)

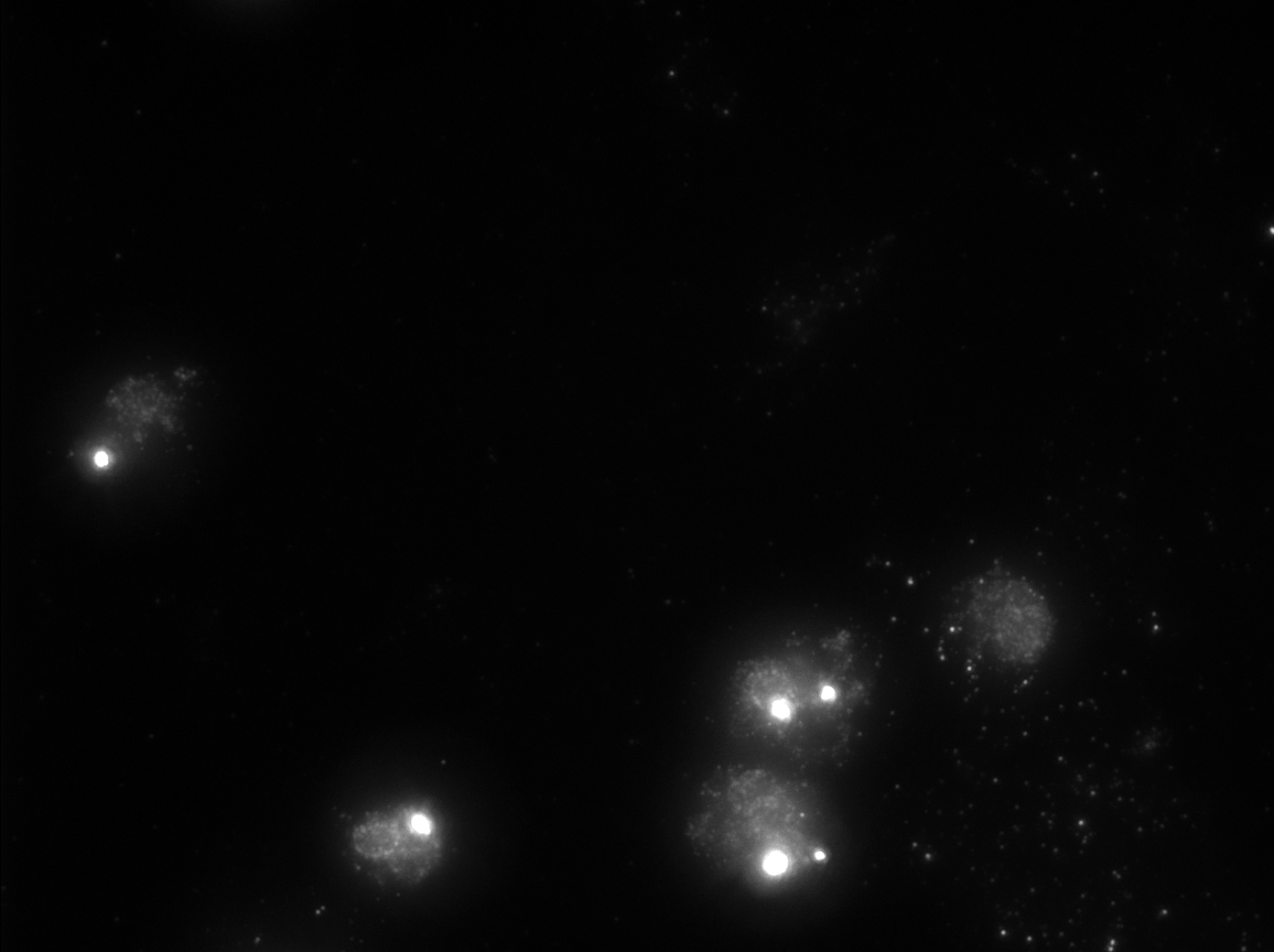

Supplement: Supplementary file 5 — Source data Fig. 1 [file 44319_2024_329_MOESM5_ESM.zip › Figure 1 Source Data/E.tif]

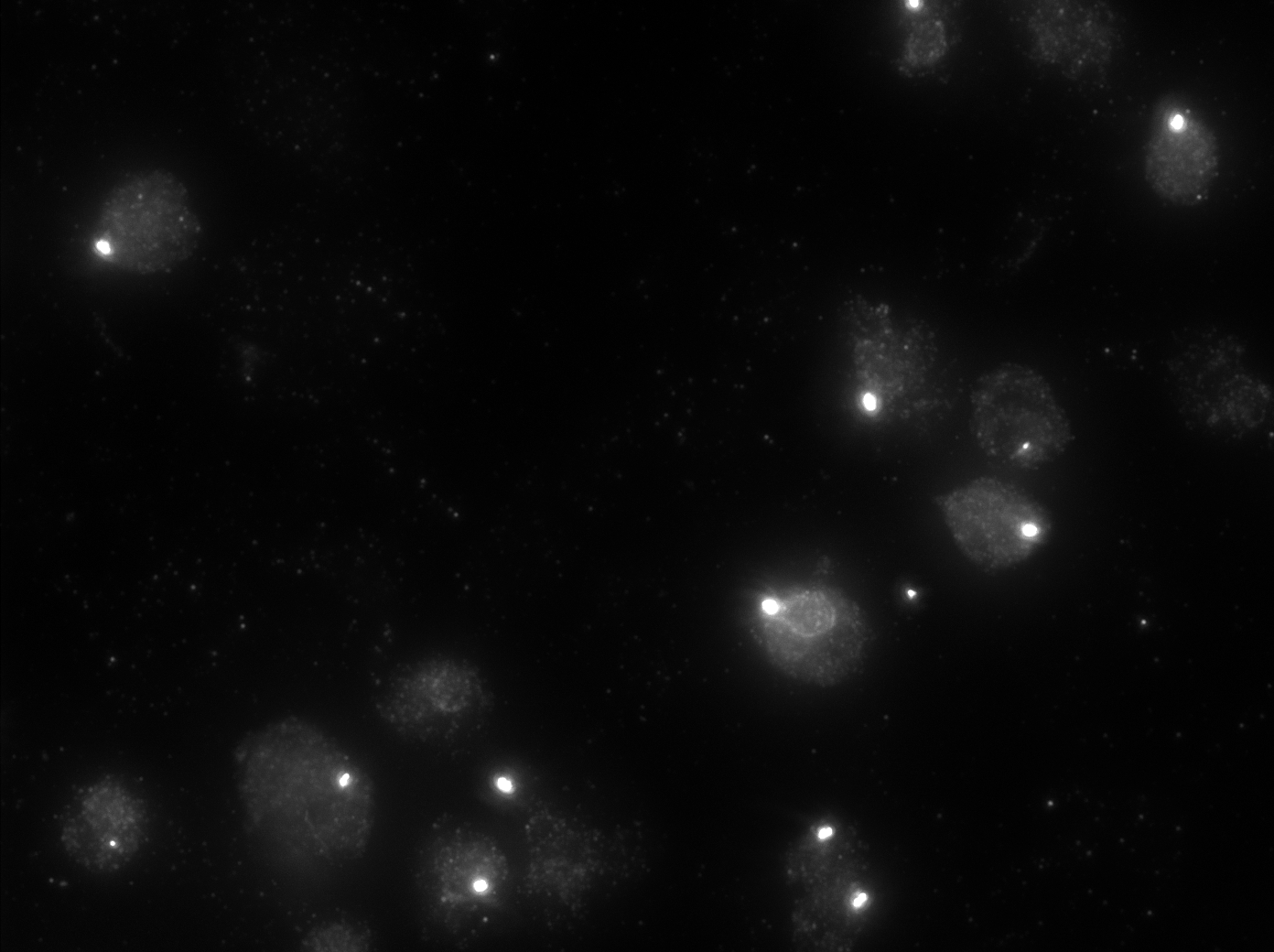

Supplement: Supplementary file 5 — Source data Fig. 1 [file 44319_2024_329_MOESM5_ESM.zip › Figure 1 Source Data/D.tif]

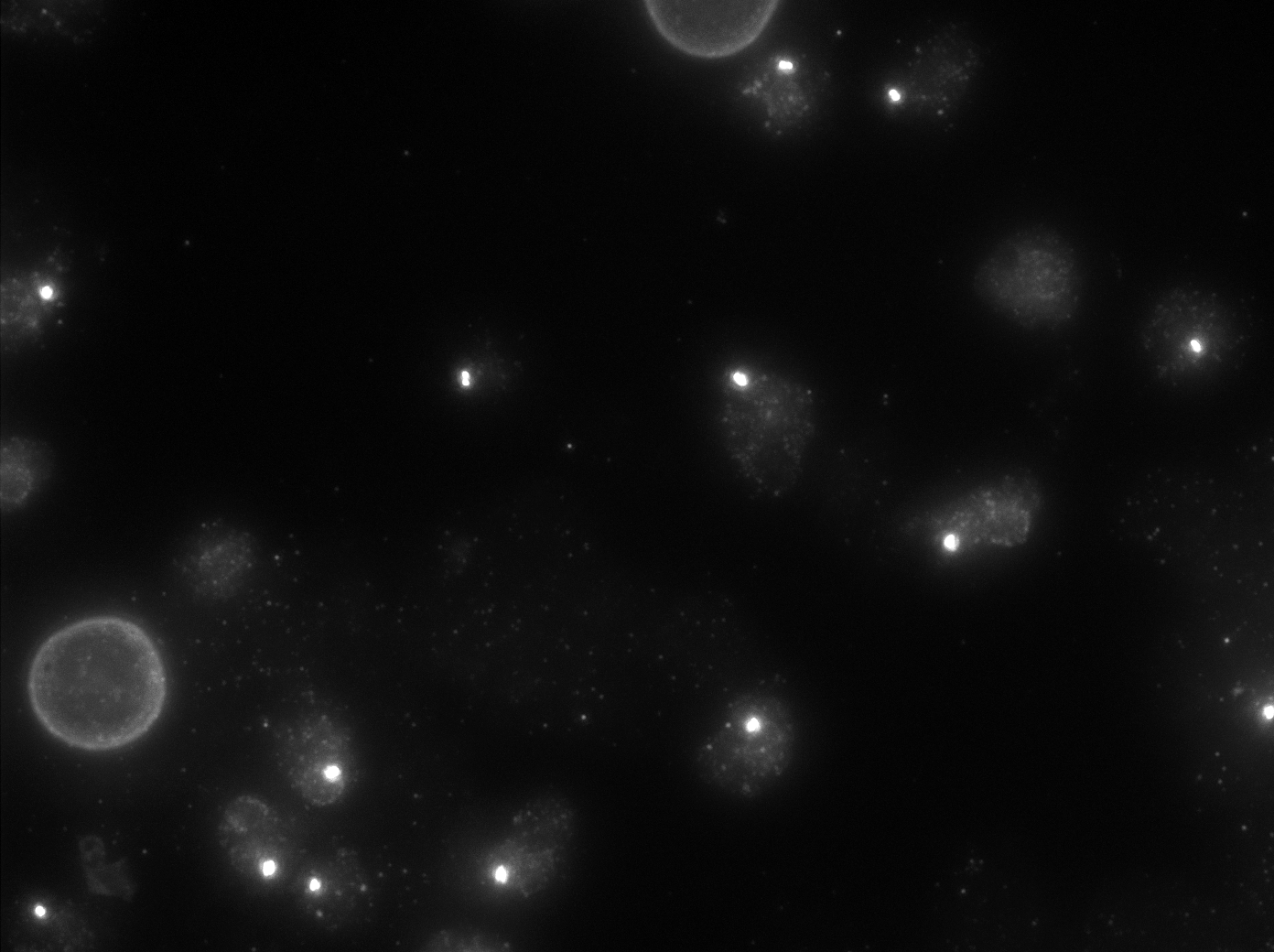

Supplement: Supplementary file 5 — Source data Fig. 1 [file 44319_2024_329_MOESM5_ESM.zip › Figure 1 Source Data/F.tif]

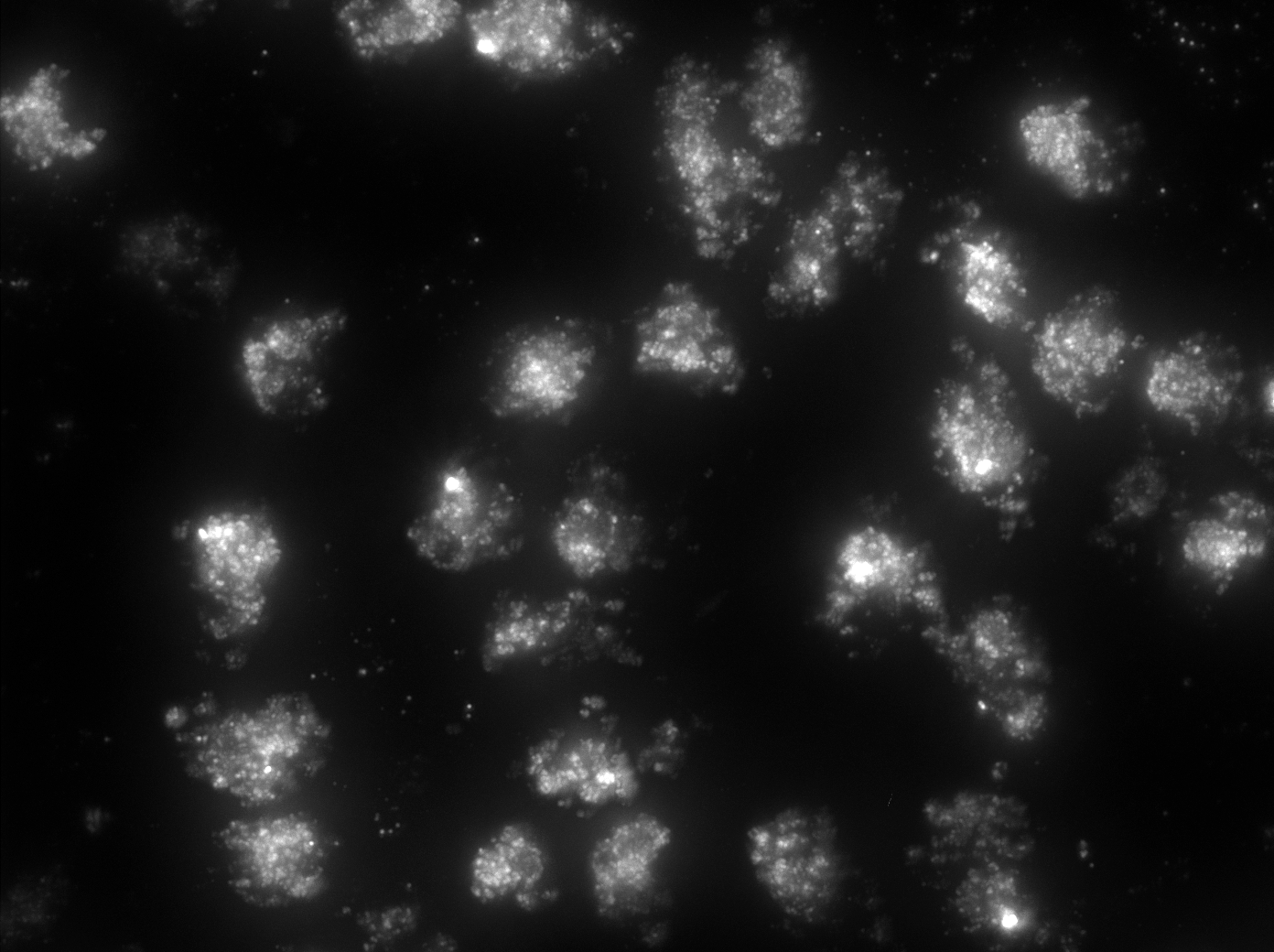

Supplement: Supplementary file 5 — Source data Fig. 1 [file 44319_2024_329_MOESM5_ESM.zip › Figure 1 Source Data/G.tif]

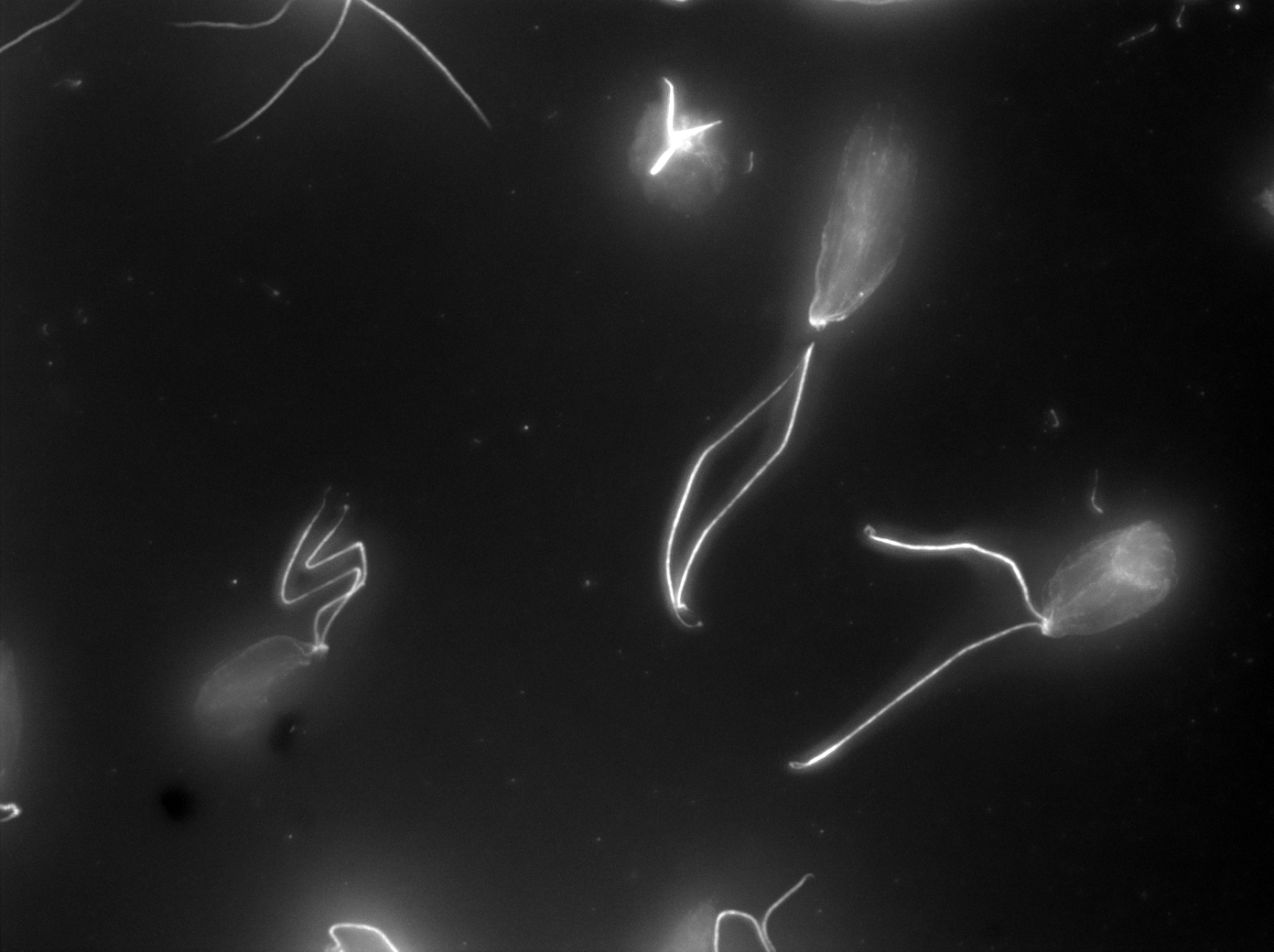

Supplement: Supplementary file 5 — Source data Fig. 1 [file 44319_2024_329_MOESM5_ESM.zip › Figure 1 Source Data/C.tif]

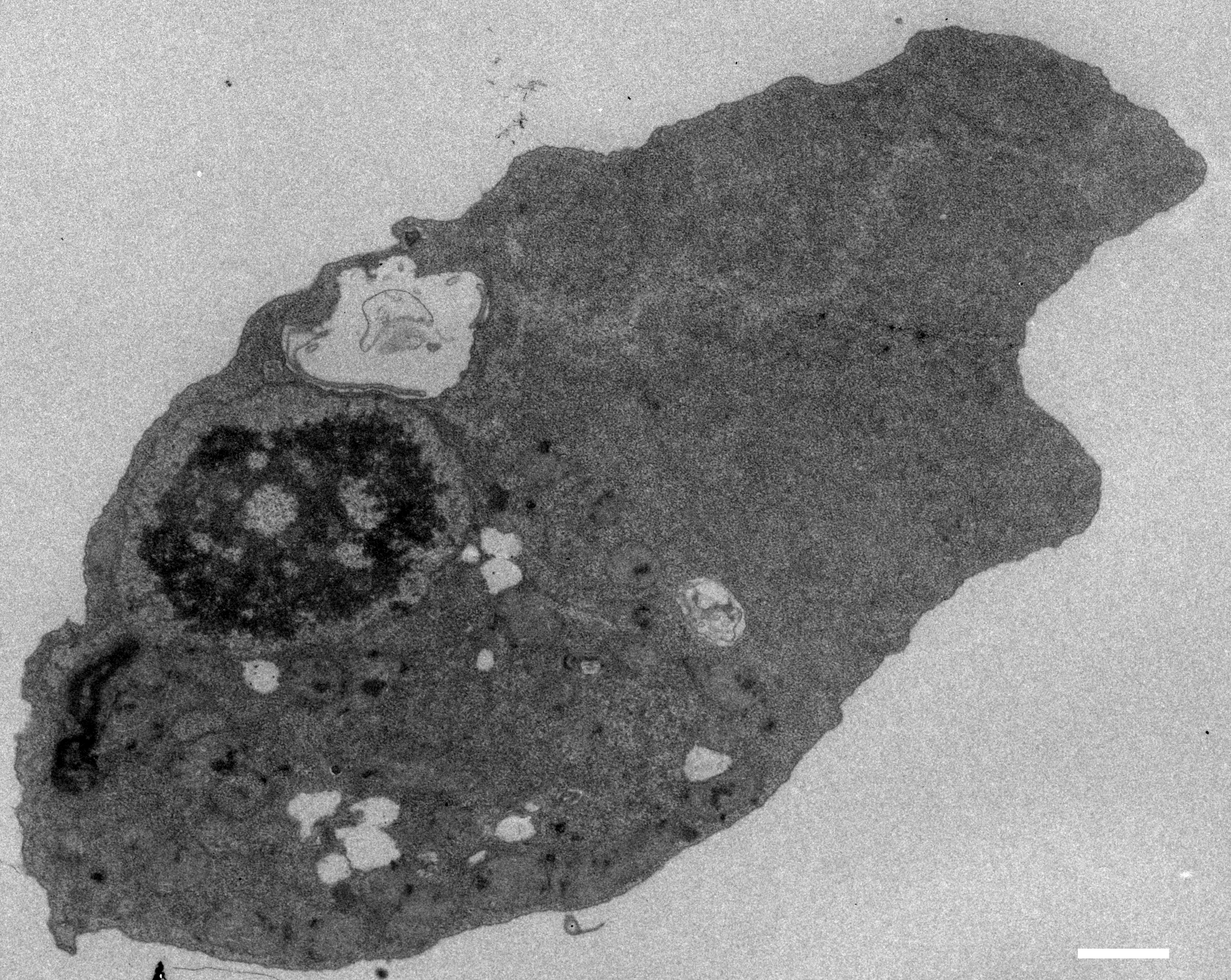

Supplement: Supplementary file 6 — Source data Fig. 2 [file 44319_2024_329_MOESM6_ESM.zip › C.tif]

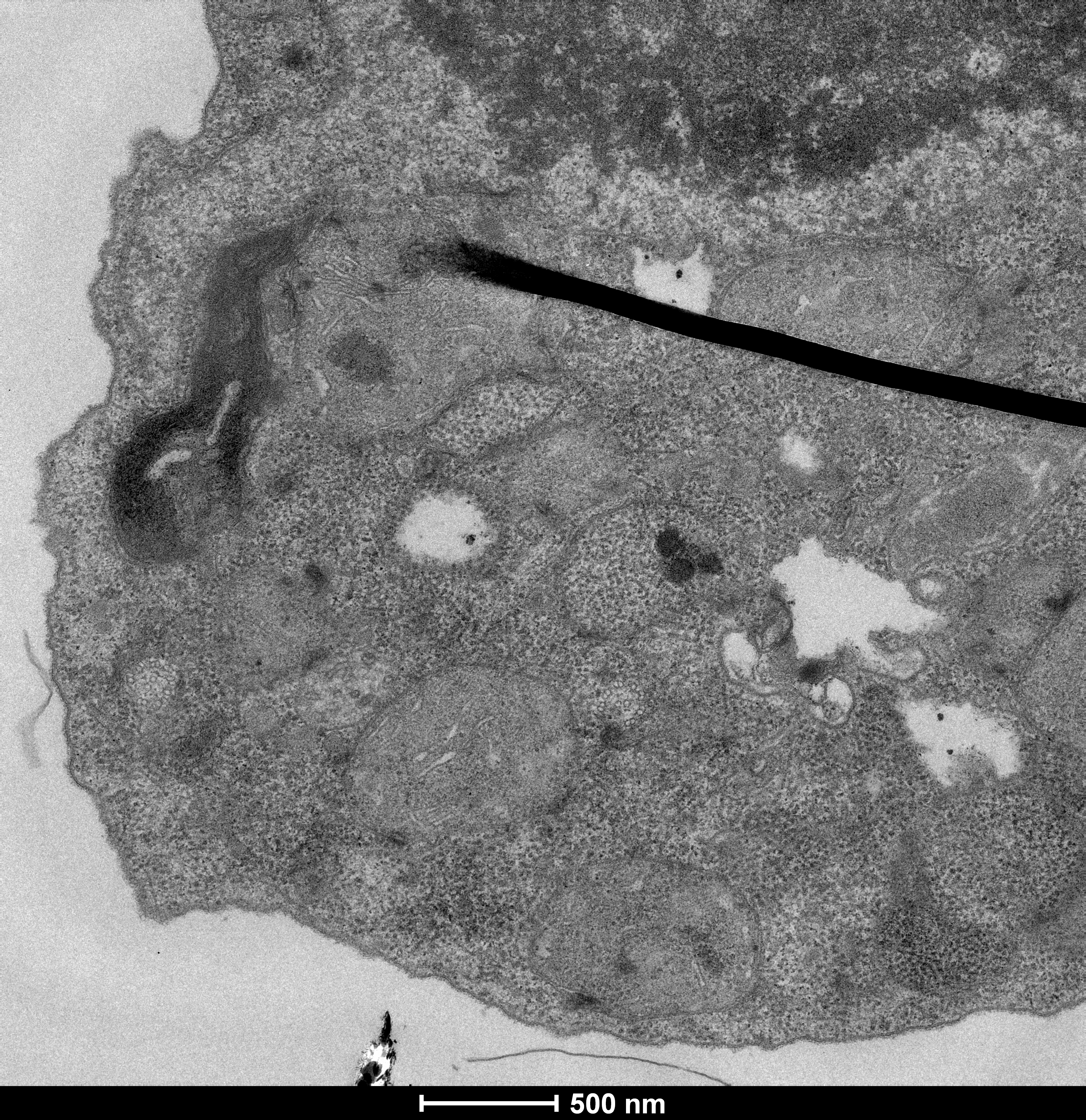

Supplement: Supplementary file 6 — Source data Fig. 2 [file 44319_2024_329_MOESM6_ESM.zip › D.tif]

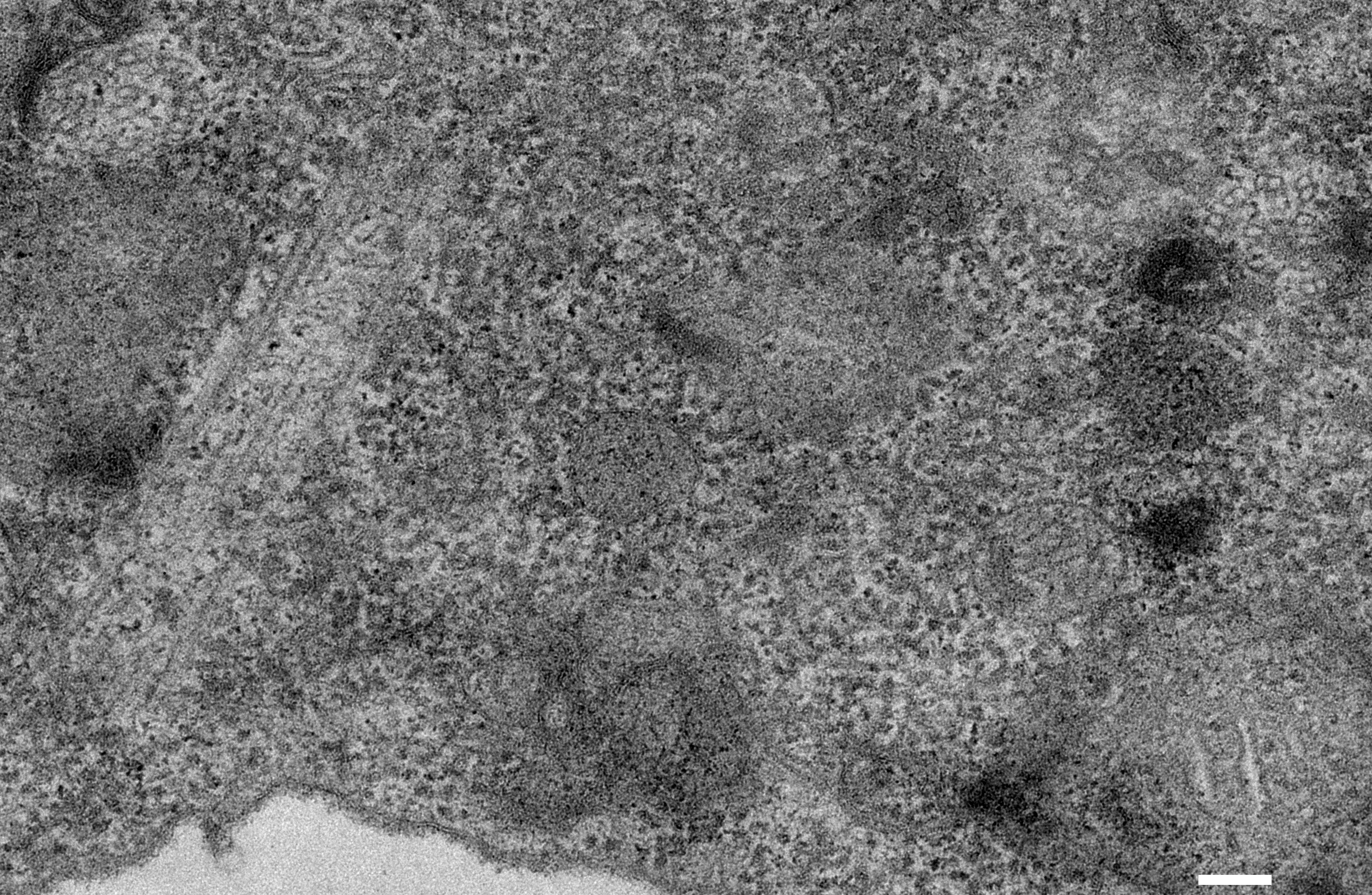

Supplement: Supplementary file 7 — Source data Fig. 3 [file 44319_2024_329_MOESM7_ESM.zip › E.tif]

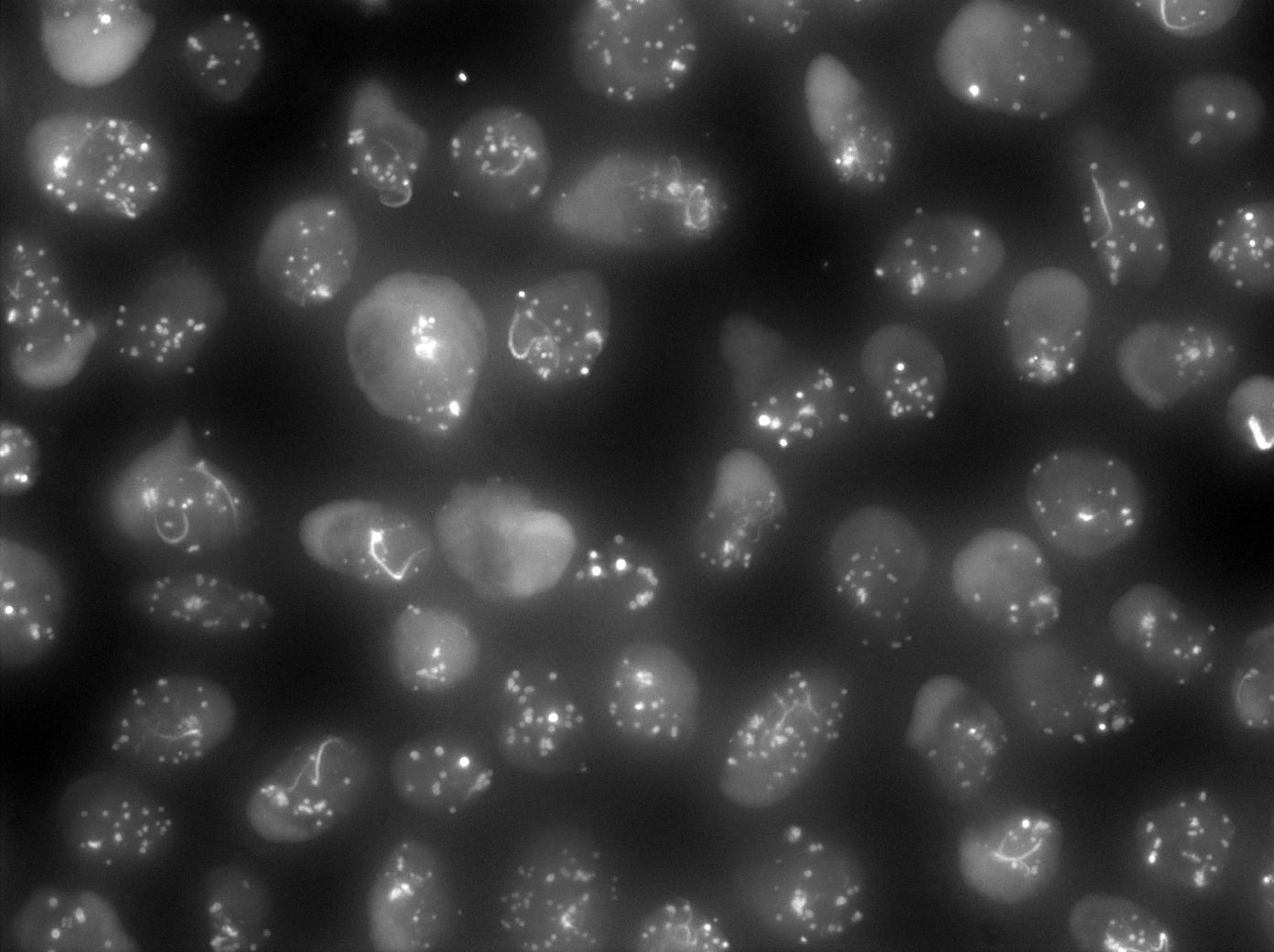

Supplement: Supplementary file 7 — Source data Fig. 3 [file 44319_2024_329_MOESM7_ESM.zip › F_top.tif]

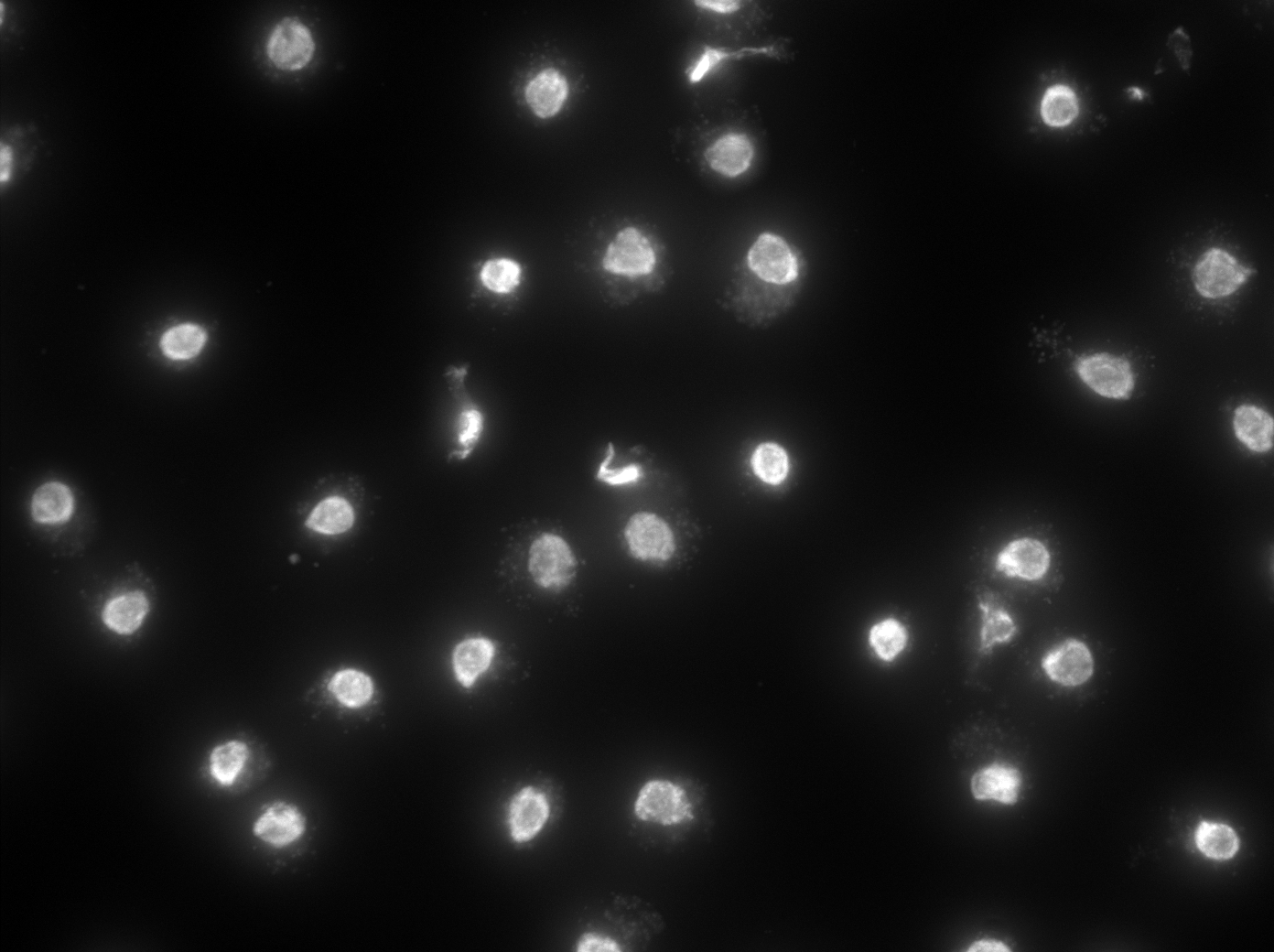

Supplement: Supplementary file 7 — Source data Fig. 3 [file 44319_2024_329_MOESM7_ESM.zip › A.tif]

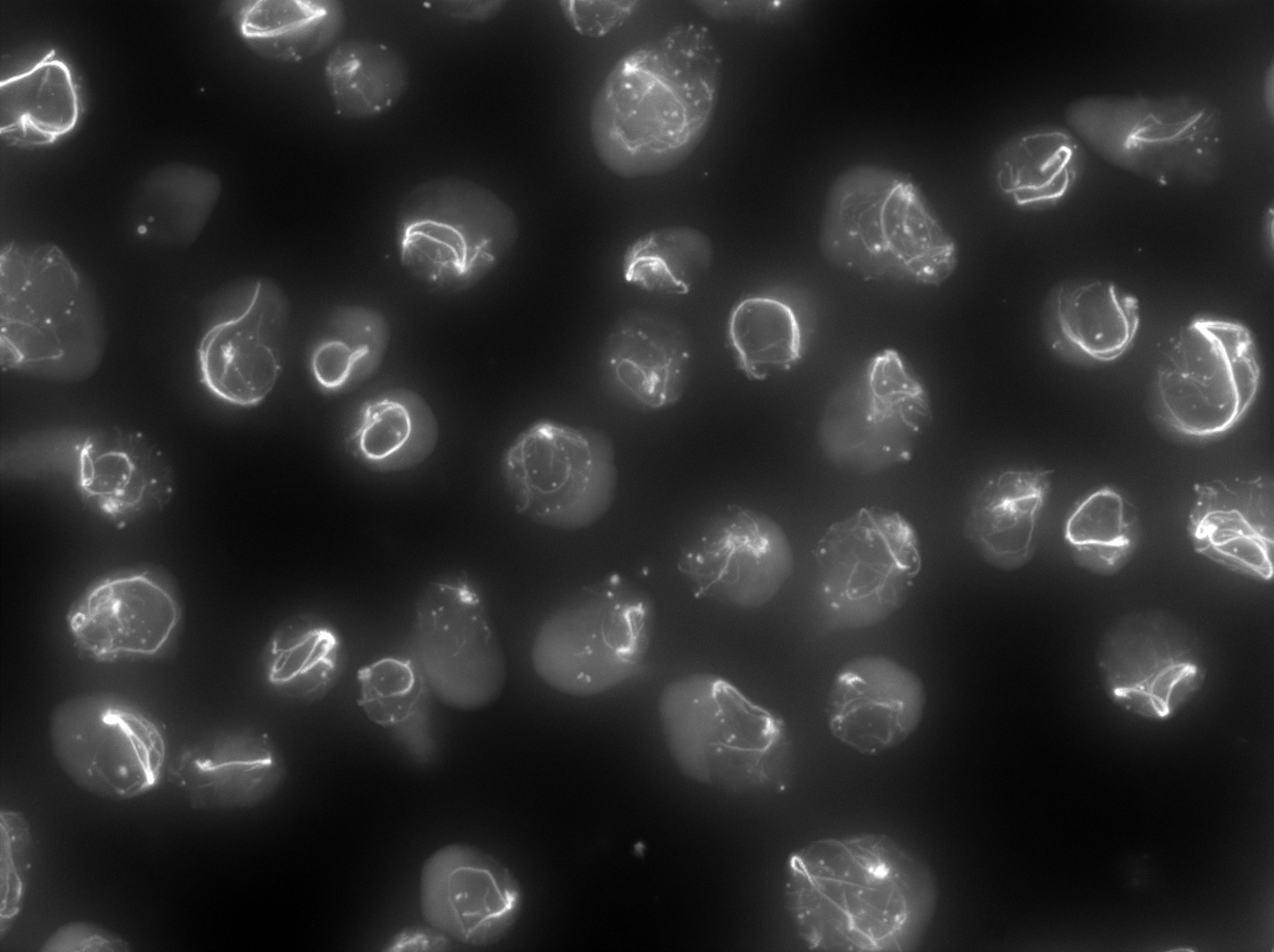

Supplement: Supplementary file 7 — Source data Fig. 3 [file 44319_2024_329_MOESM7_ESM.zip › F_bottom.tif]

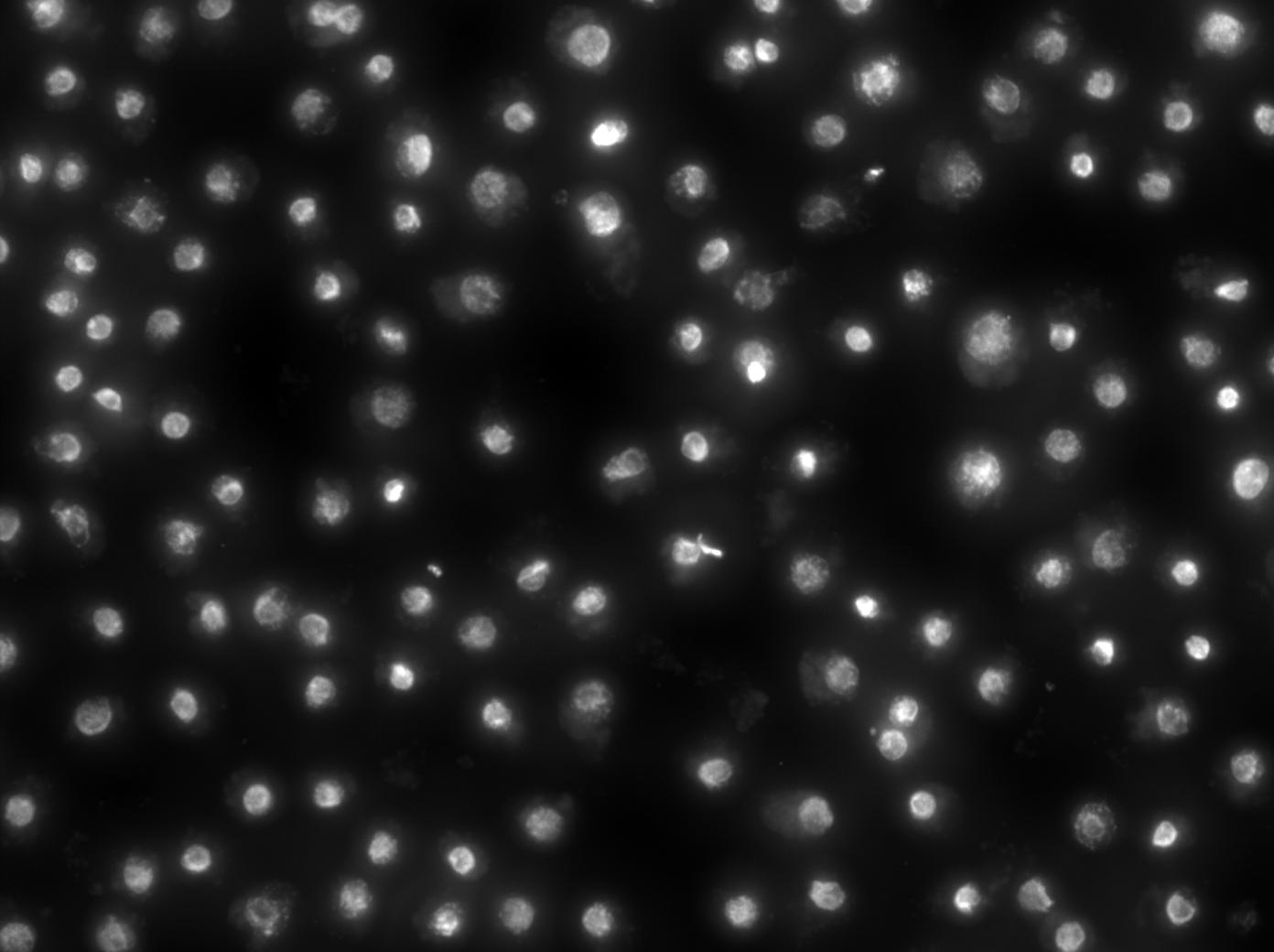

Supplement: Supplementary file 8 — Source data Fig. 4 [file 44319_2024_329_MOESM8_ESM.zip › Figure 4 Source Data/H_right.tif]

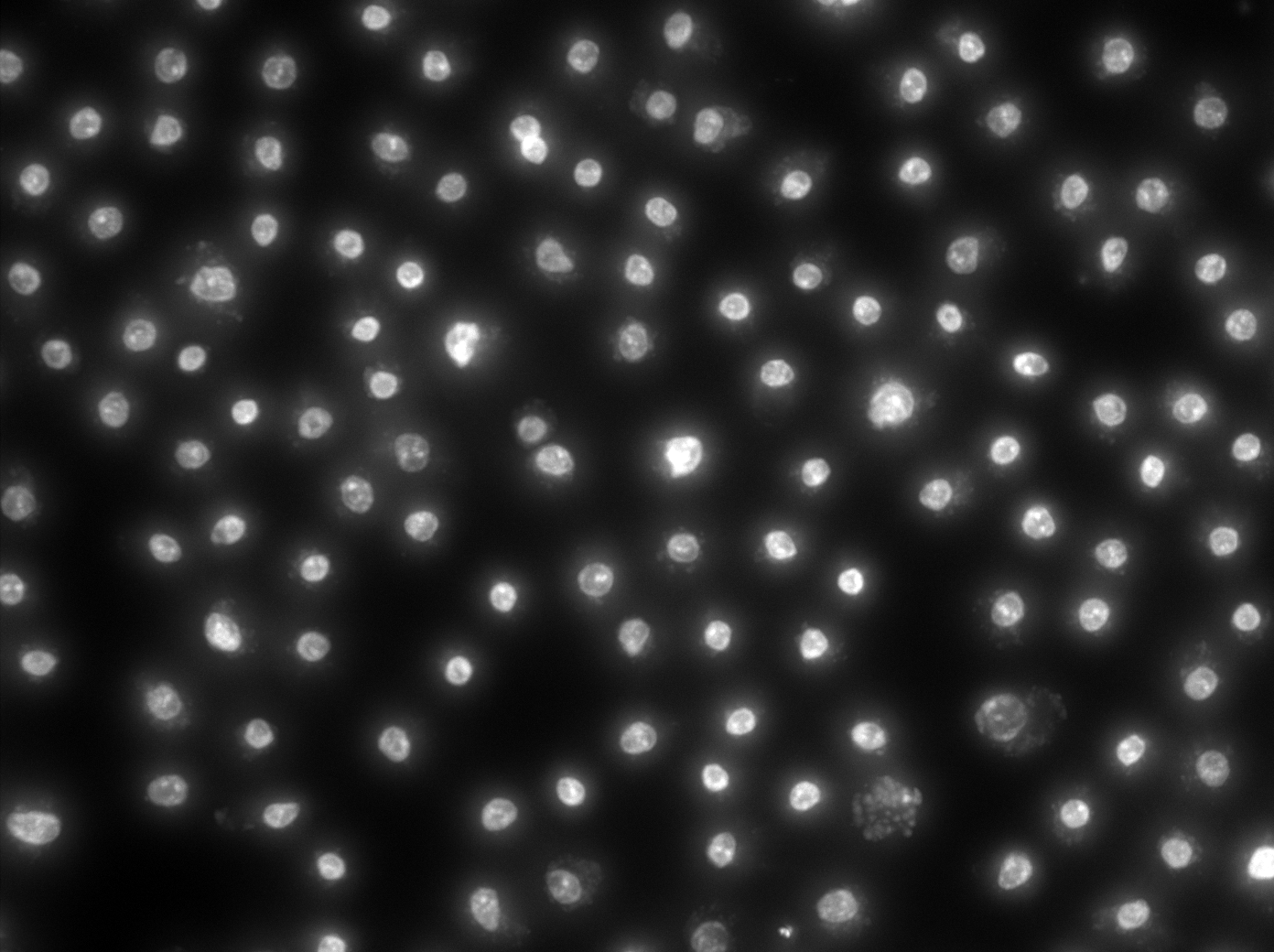

Supplement: Supplementary file 8 — Source data Fig. 4 [file 44319_2024_329_MOESM8_ESM.zip › Figure 4 Source Data/F_left.tif]

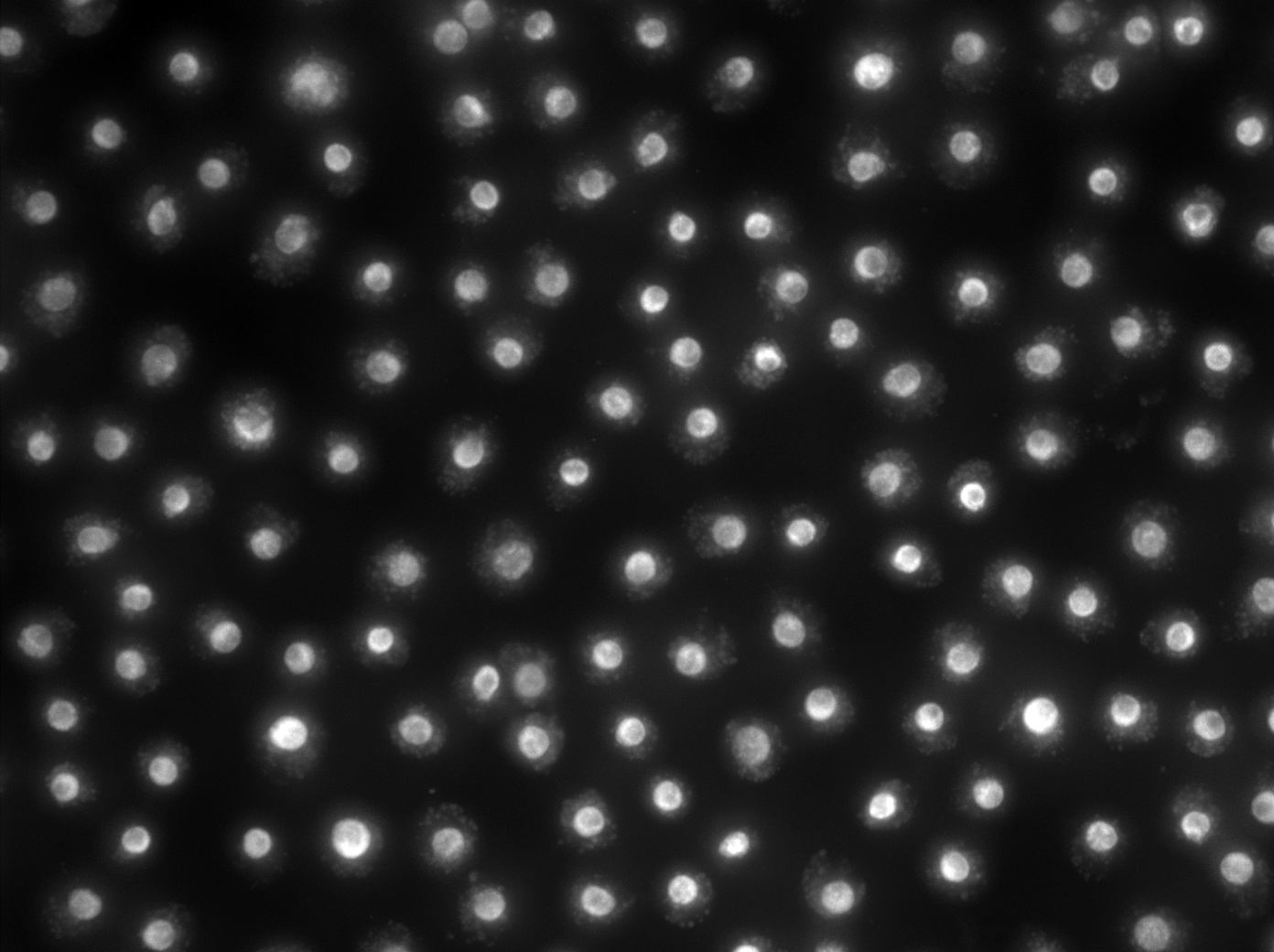

Supplement: Supplementary file 8 — Source data Fig. 4 [file 44319_2024_329_MOESM8_ESM.zip › Figure 4 Source Data/D_right.tif]

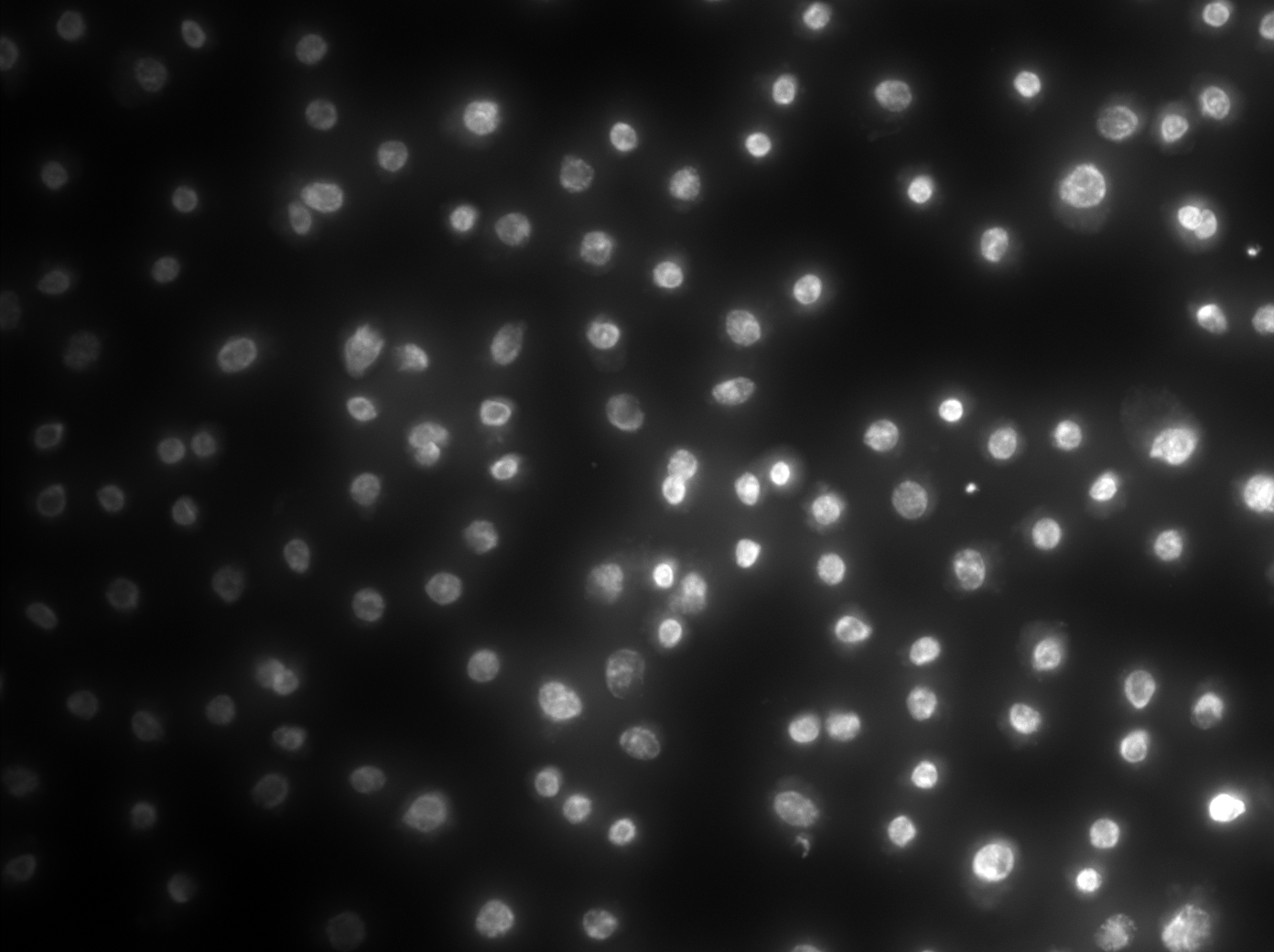

Supplement: Supplementary file 8 — Source data Fig. 4 [file 44319_2024_329_MOESM8_ESM.zip › Figure 4 Source Data/H_left.tif]

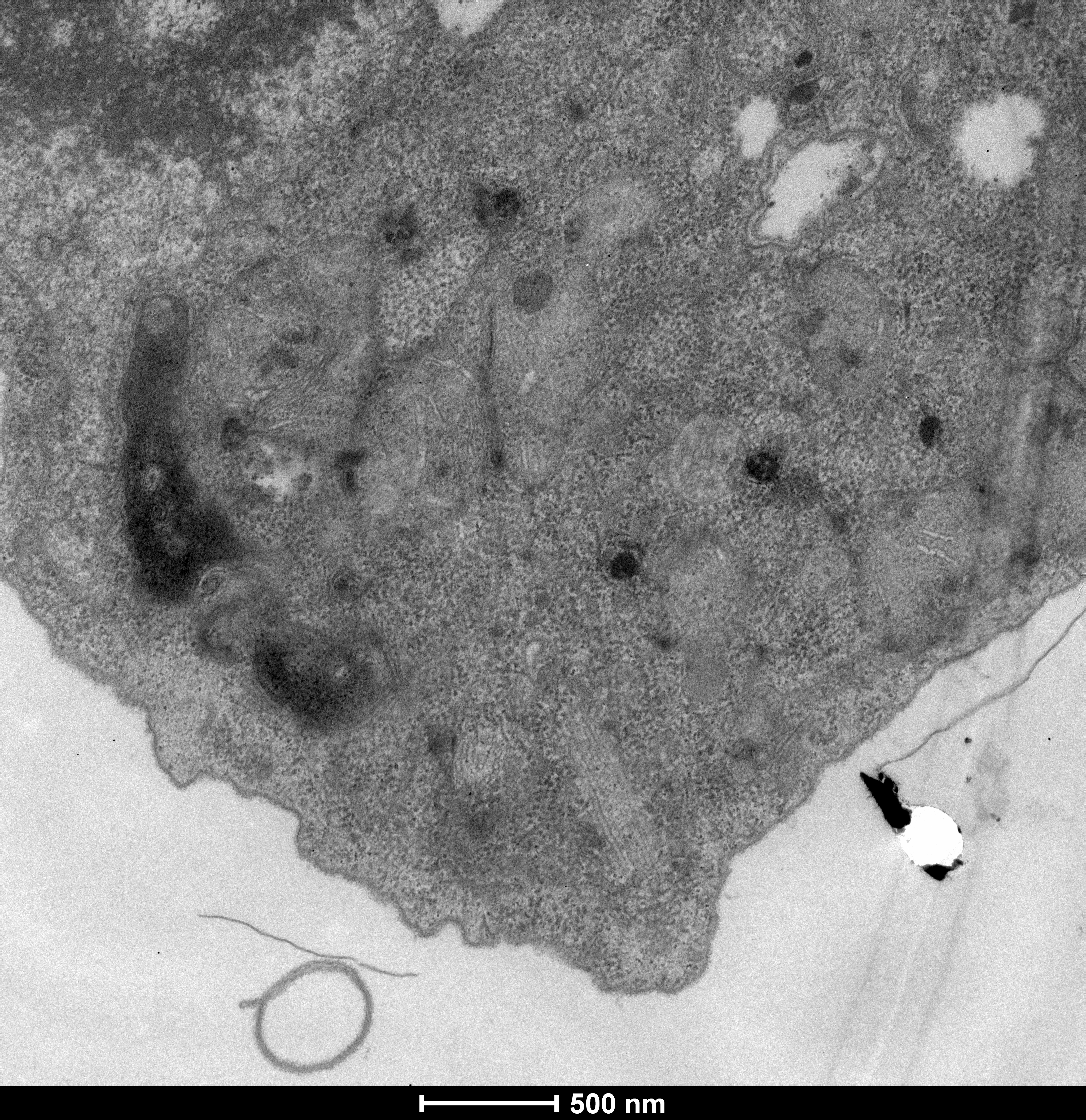

Supplement: Supplementary file 8 — Source data Fig. 4 [file 44319_2024_329_MOESM8_ESM.zip › Figure 4 Source Data/B.tif]

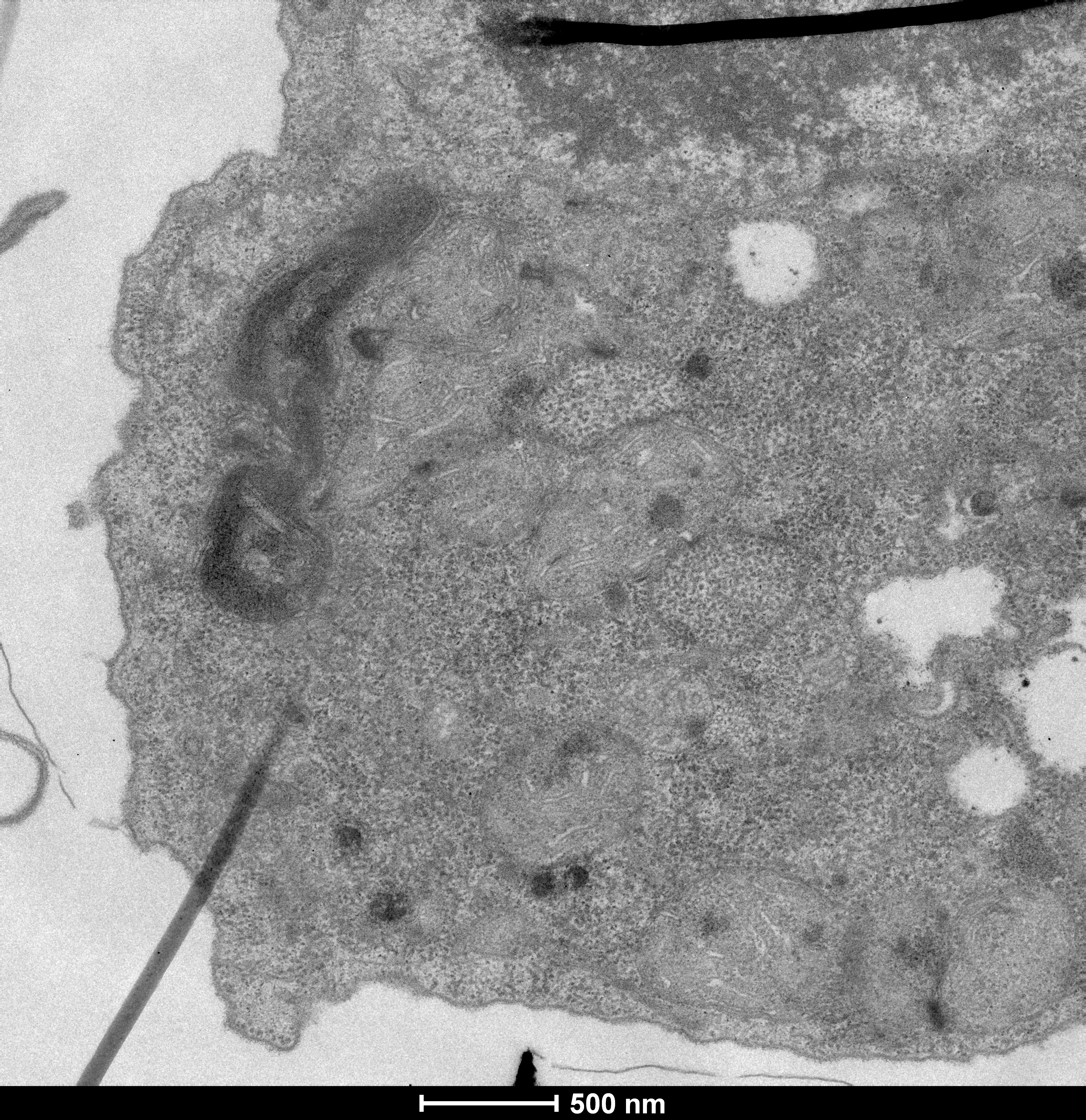

Supplement: Supplementary file 8 — Source data Fig. 4 [file 44319_2024_329_MOESM8_ESM.zip › Figure 4 Source Data/A.tif]

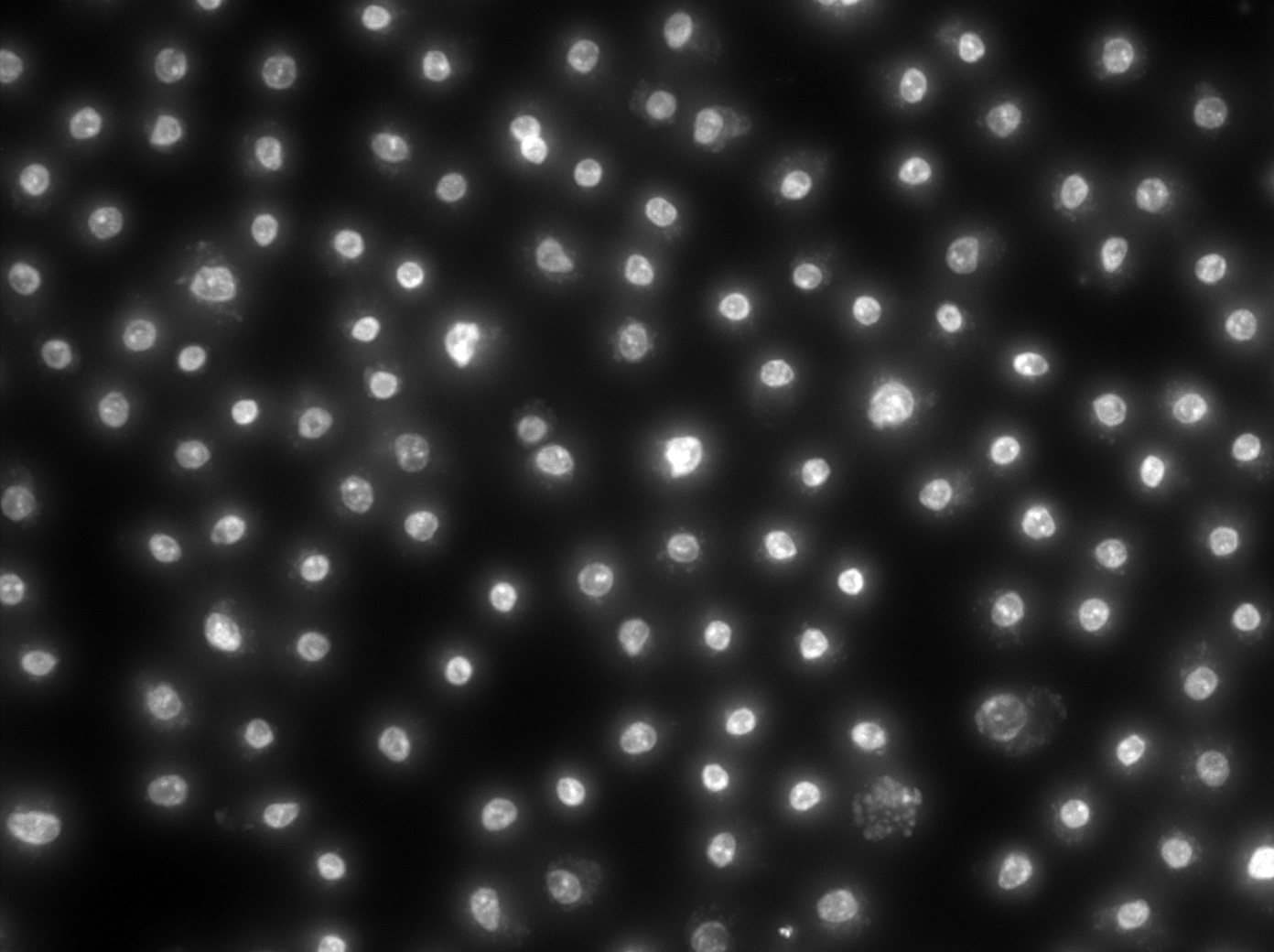

Supplement: Supplementary file 8 — Source data Fig. 4 [file 44319_2024_329_MOESM8_ESM.zip › Figure 4 Source Data/D_left.tif]

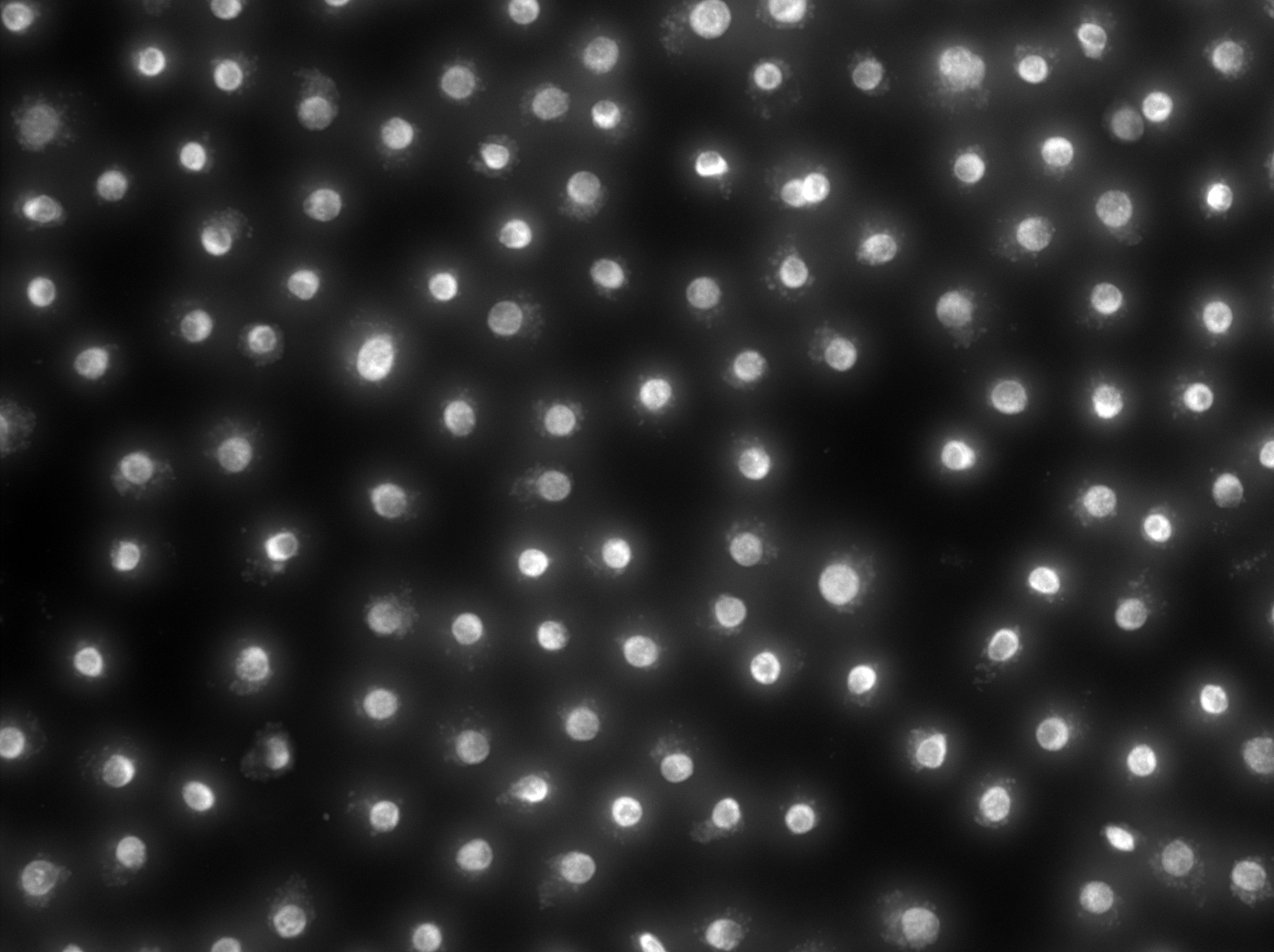

Supplement: Supplementary file 8 — Source data Fig. 4 [file 44319_2024_329_MOESM8_ESM.zip › Figure 4 Source Data/F_right.tif]

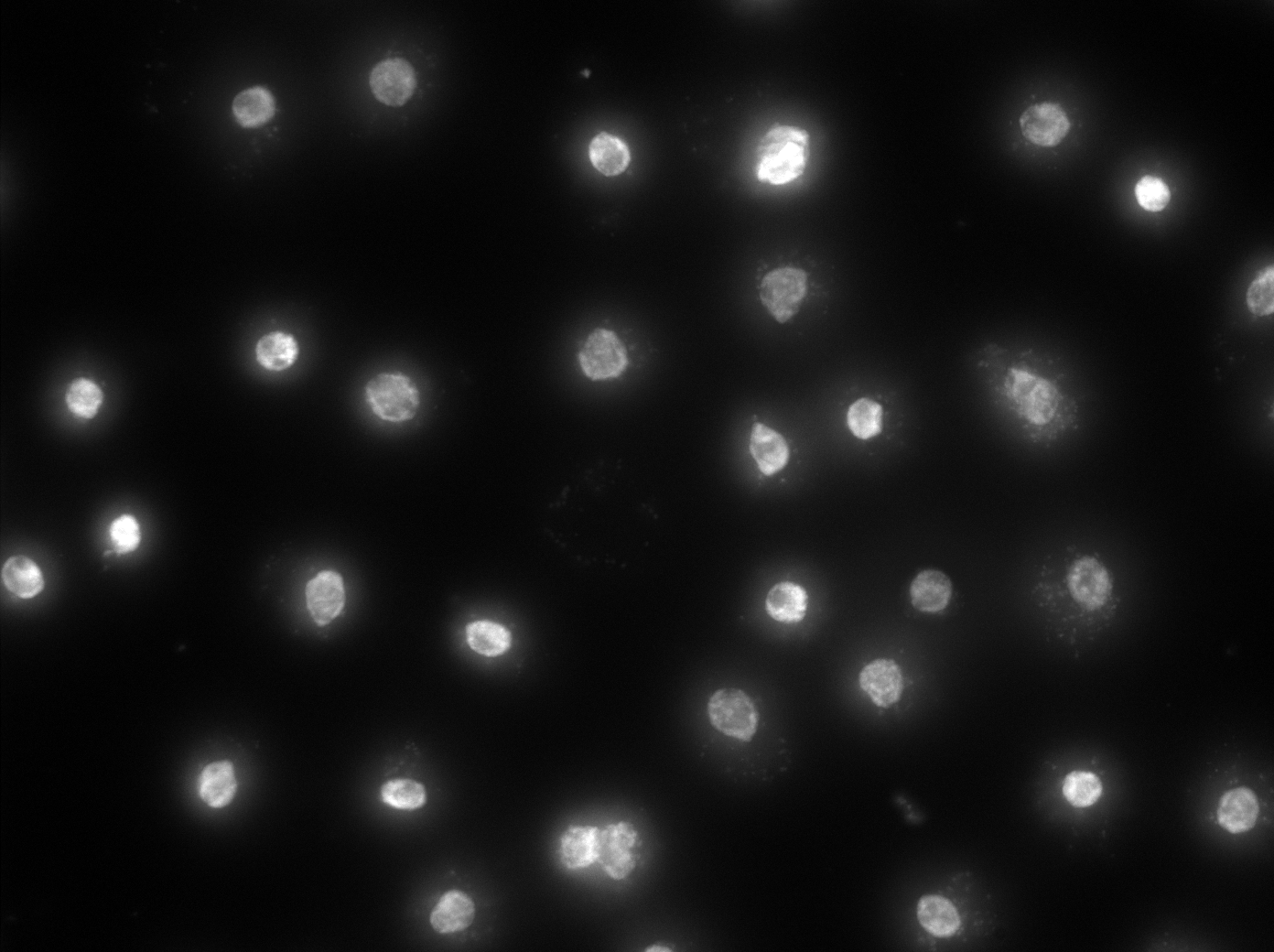

Supplement: Supplementary file 9 — Source data Fig. 5 [file 44319_2024_329_MOESM9_ESM.zip › D_bottom.tif]

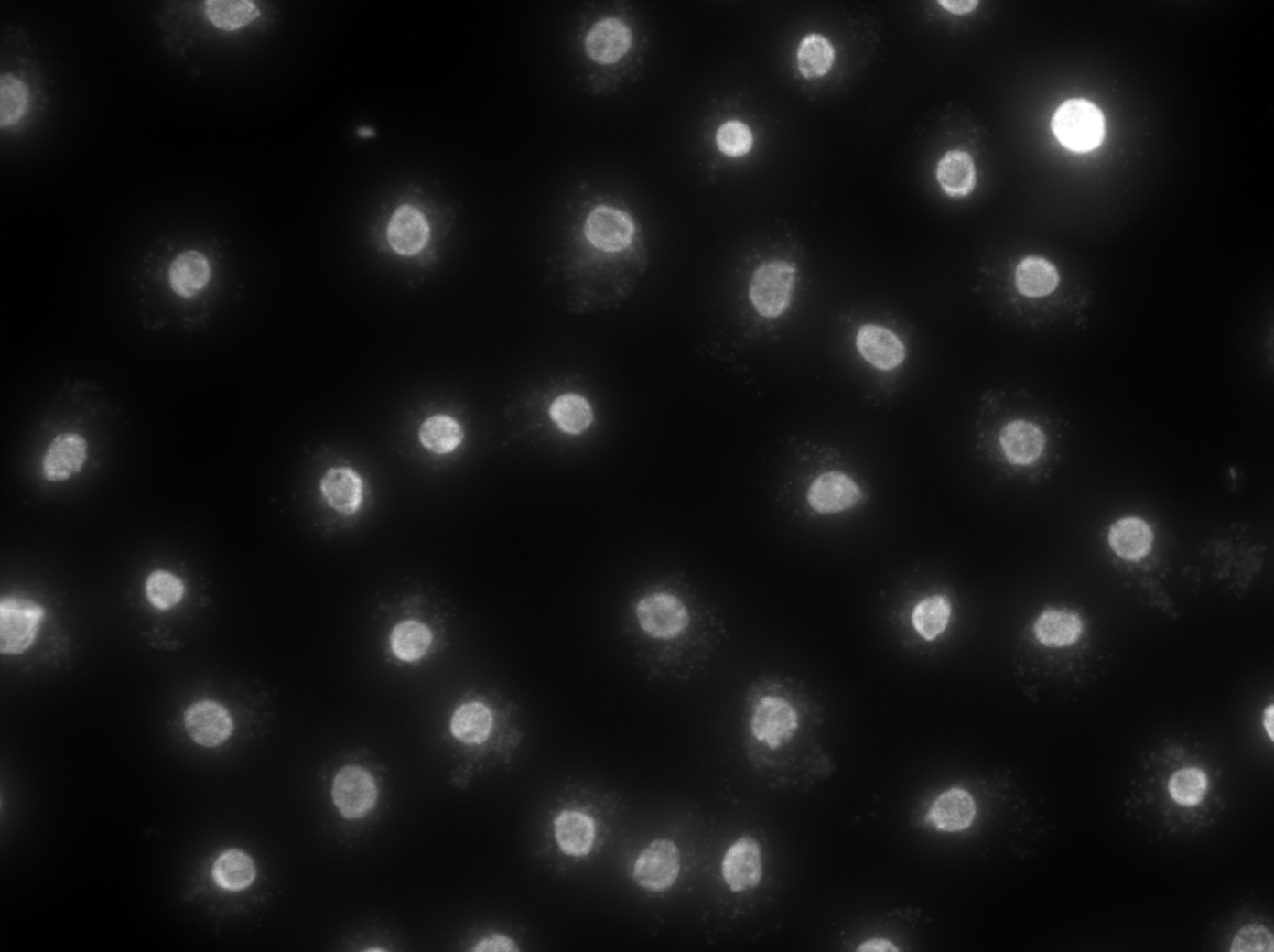

Supplement: Supplementary file 9 — Source data Fig. 5 [file 44319_2024_329_MOESM9_ESM.zip › D_top.tif]

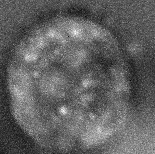

Supplement: Supplementary file 9 — Source data Fig. 5 [file 44319_2024_329_MOESM9_ESM.zip › F.tif]

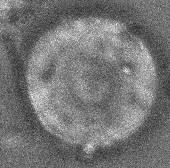

Supplement: Supplementary file 9 — Source data Fig. 5 [file 44319_2024_329_MOESM9_ESM.zip › G.tif]

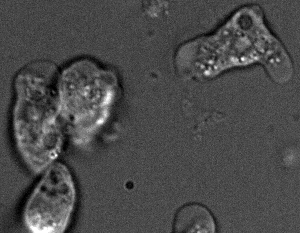

Supplement: Supplementary file 9 — Source data Fig. 5 [file 44319_2024_329_MOESM9_ESM.zip › I.tif]

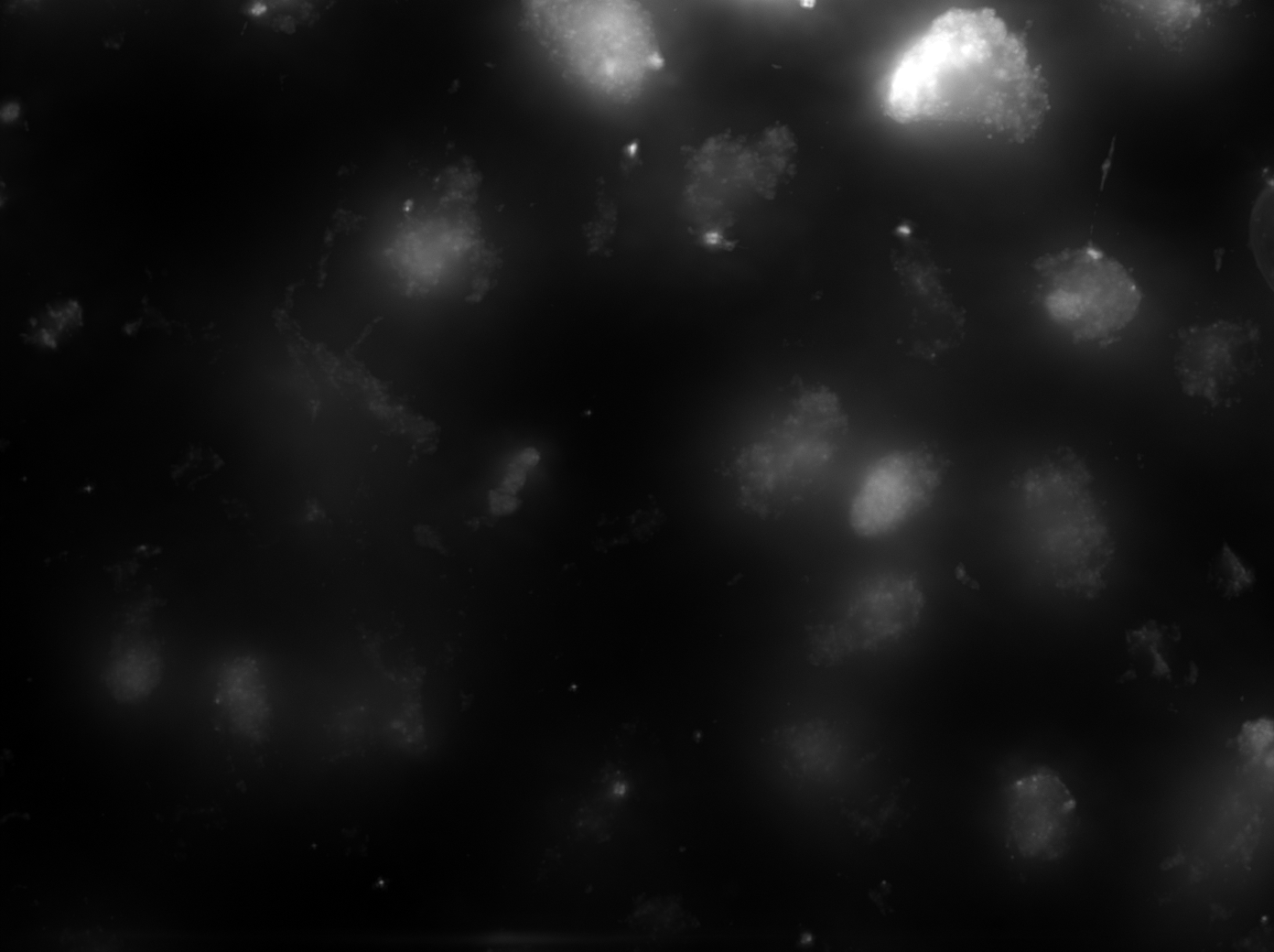

Supplement: Supplementary file 10 — Source data Fig. 6 [file 44319_2024_329_MOESM10_ESM.zip › F.tif]

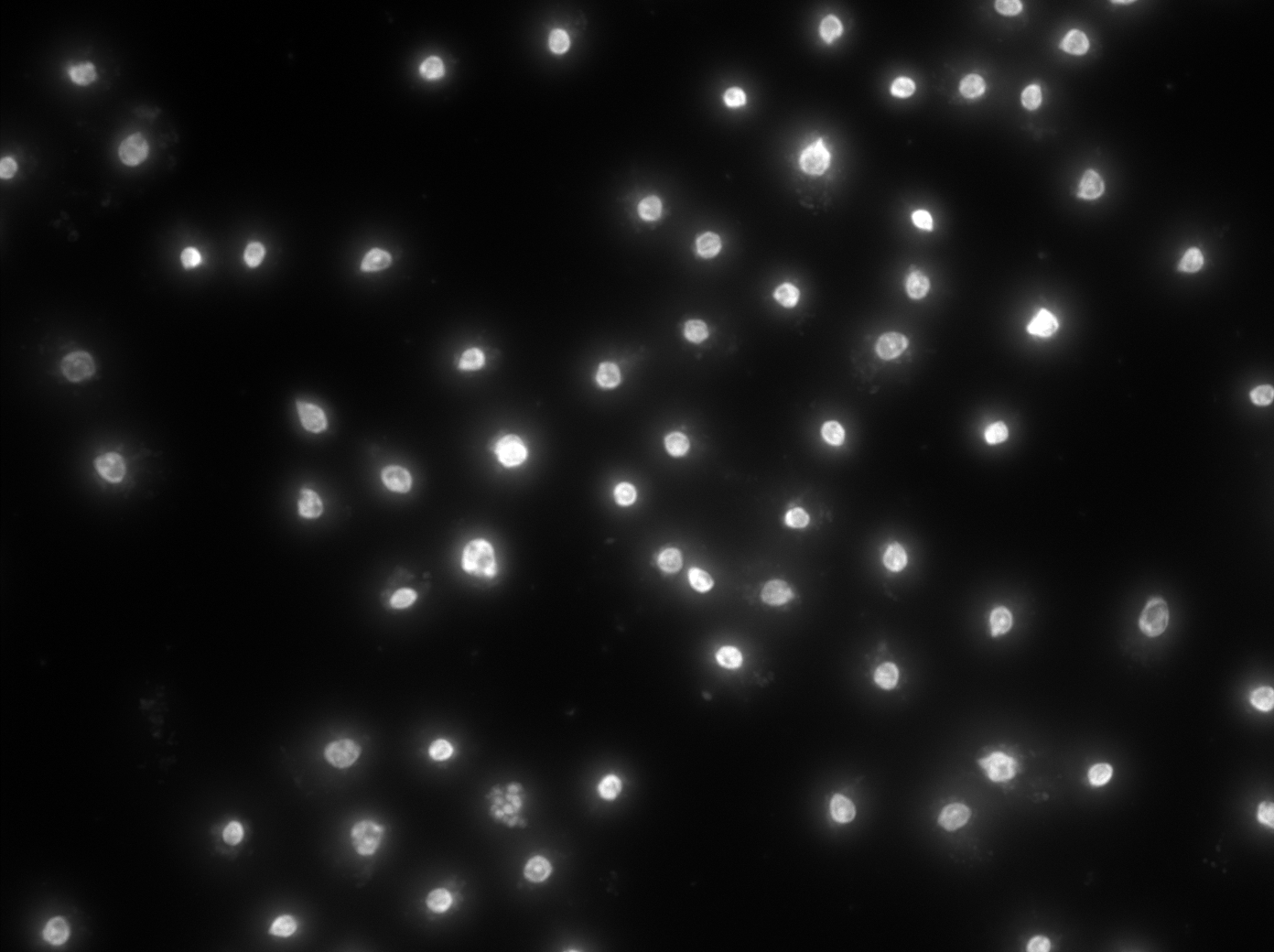

Supplement: Supplementary file 10 — Source data Fig. 6 [file 44319_2024_329_MOESM10_ESM.zip › C.tif]

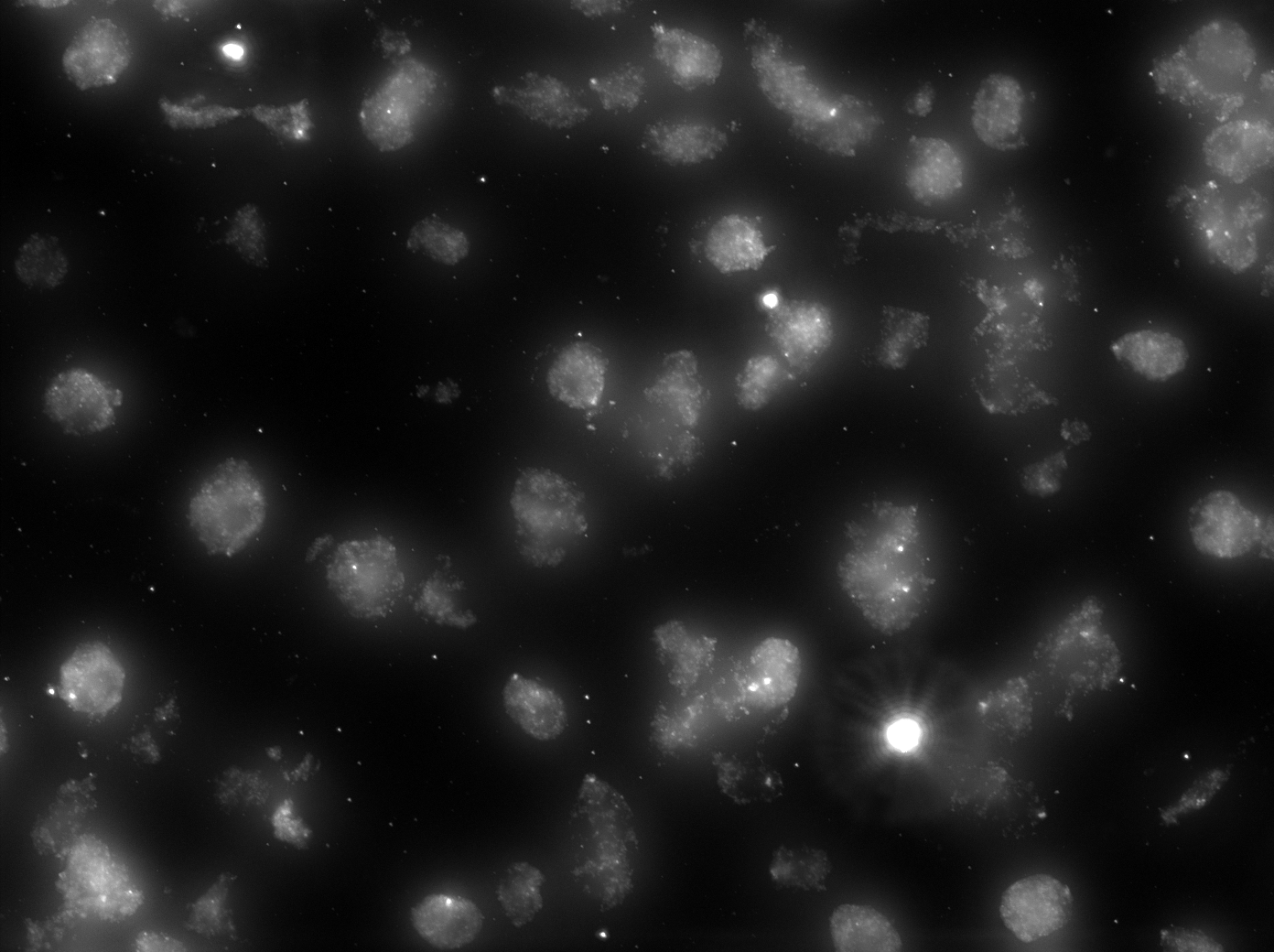

Supplement: Supplementary file 10 — Source data Fig. 6 [file 44319_2024_329_MOESM10_ESM.zip › E.tif]

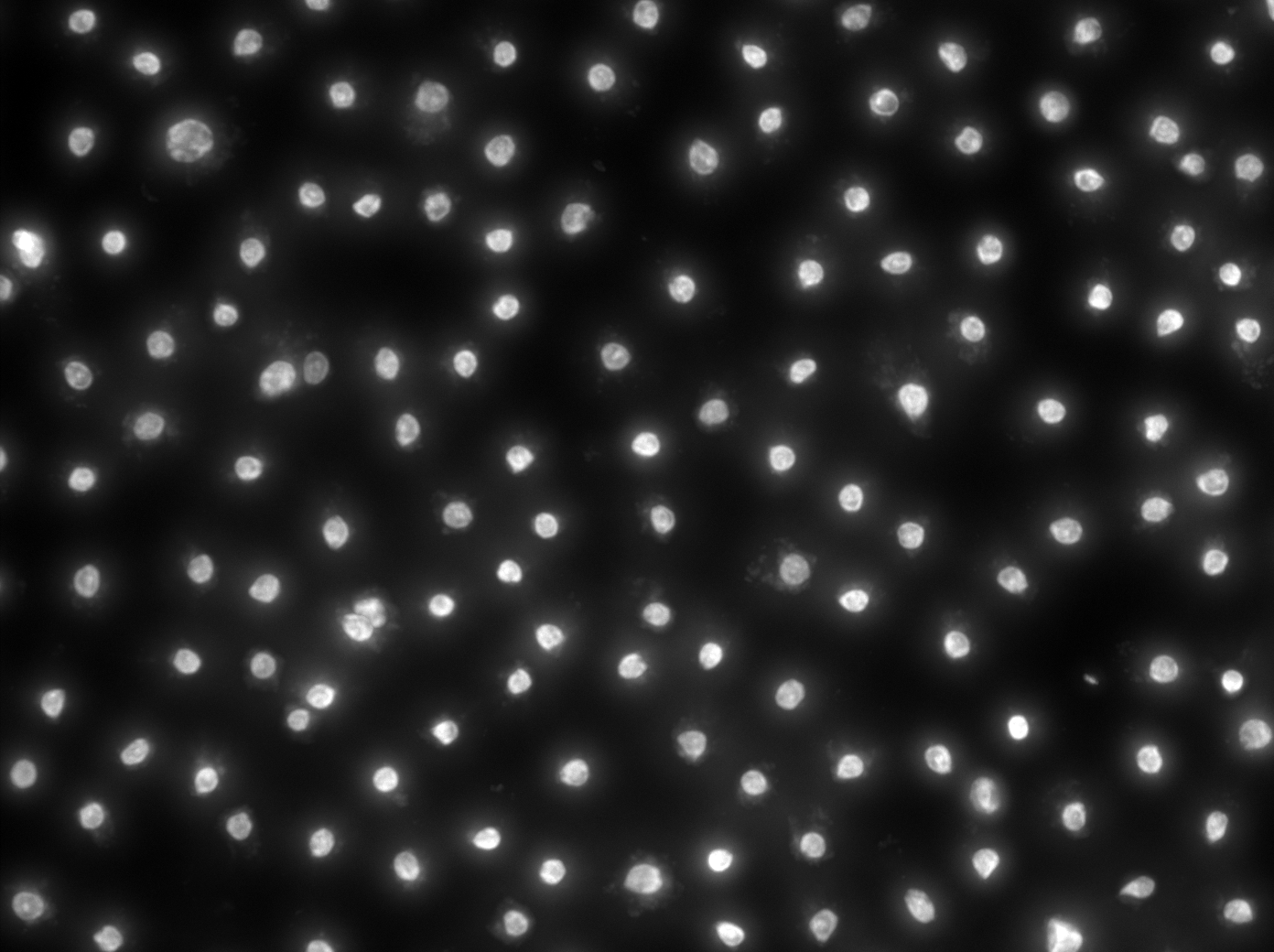

Supplement: Supplementary file 10 — Source data Fig. 6 [file 44319_2024_329_MOESM10_ESM.zip › D.tif]
