# Supplementary material for: Mechanisms of axoneme and centriole elimination in Naegleria gruberi
Source: EMBO Rep. 2024 Dec 2;26(2):385–406. doi: 10.1038/s44319-024-00329-w (PMC11772885; doi:10.1038/s44319-024-00329-w)
Supplement: Supplementary file 11 — Expanded View Figures [file 44319_2024_329_MOESM11_ESM.pdf]

Expanded View Figures

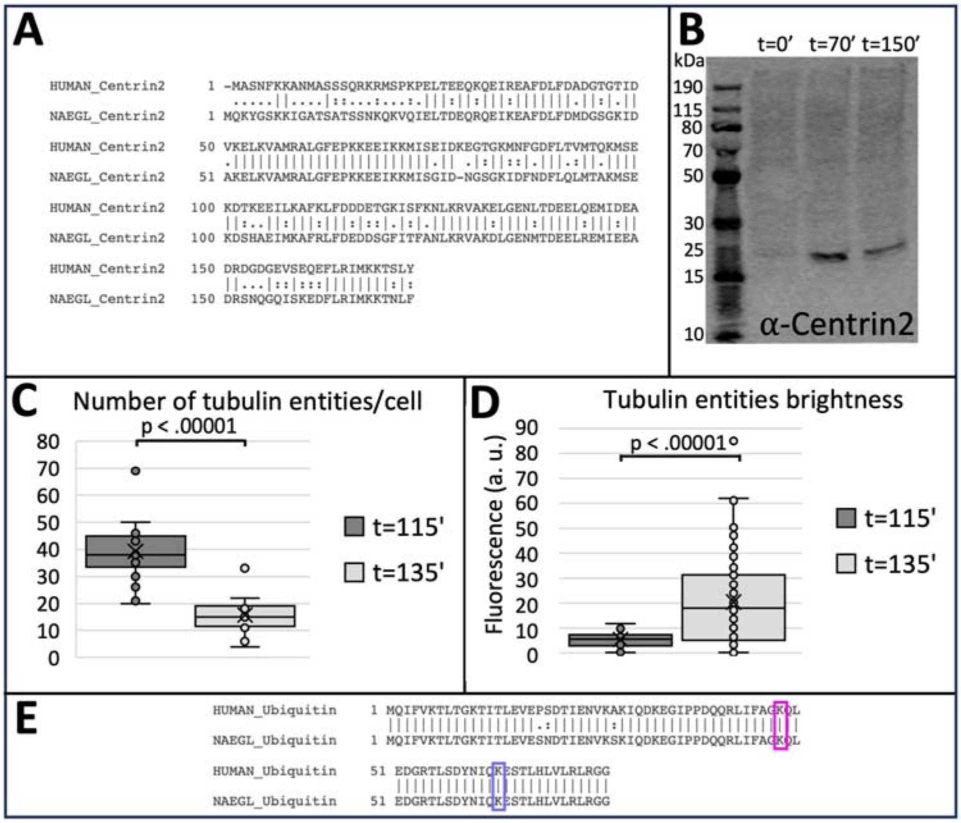

Figure EV1. Changes in amount and brightness of α/β tubulin spots after axoneme severing.

(**A**) ClustalW alignment of human and *Naegleria* Centrin2. (**B**) Western blot with Centrin antibodies raised against the human protein Centrin-2 on *Naegleria* whole cell lysates harvested at $t = 0$, 70 and 150 min. Note single band at expected size appearing at $t = 70$ min. (**C**, **D**) Quantification of number (**A**, $N = 21$ cell per time point) and brightness (**B**, $N = 100$ tubulin spots per time point) of α/β tubulin spots per cell at $t = 115$ min and $t = 135$ min after transformation onset, as indicated. Cells were heat shocked from $t = 95$ min until $t = 115$ min. Boxes represent the interquartile range (IQR), containing the middle 50% of the data. Line represents the median, the cross the mean. Whiskers extend from the first (Q1) and third quartile (Q3) down and up to the smallest and largest value within 1.5 times the IQR below Q1 and above Q3. All data points including outliers are presented. Pairwise comparison by a two-tailed Student's t-tests was conducted, with the resulting *p* values of 3.72E−10 for (**C**) and 1E−15 for (**D**). (**E**) ClustalW alignment of human and *Naegleria* ubiquitin. The K48 (magenta) and K63 (blue) poly-ubiquitinated residues are highlighted.

 © The Author(s)

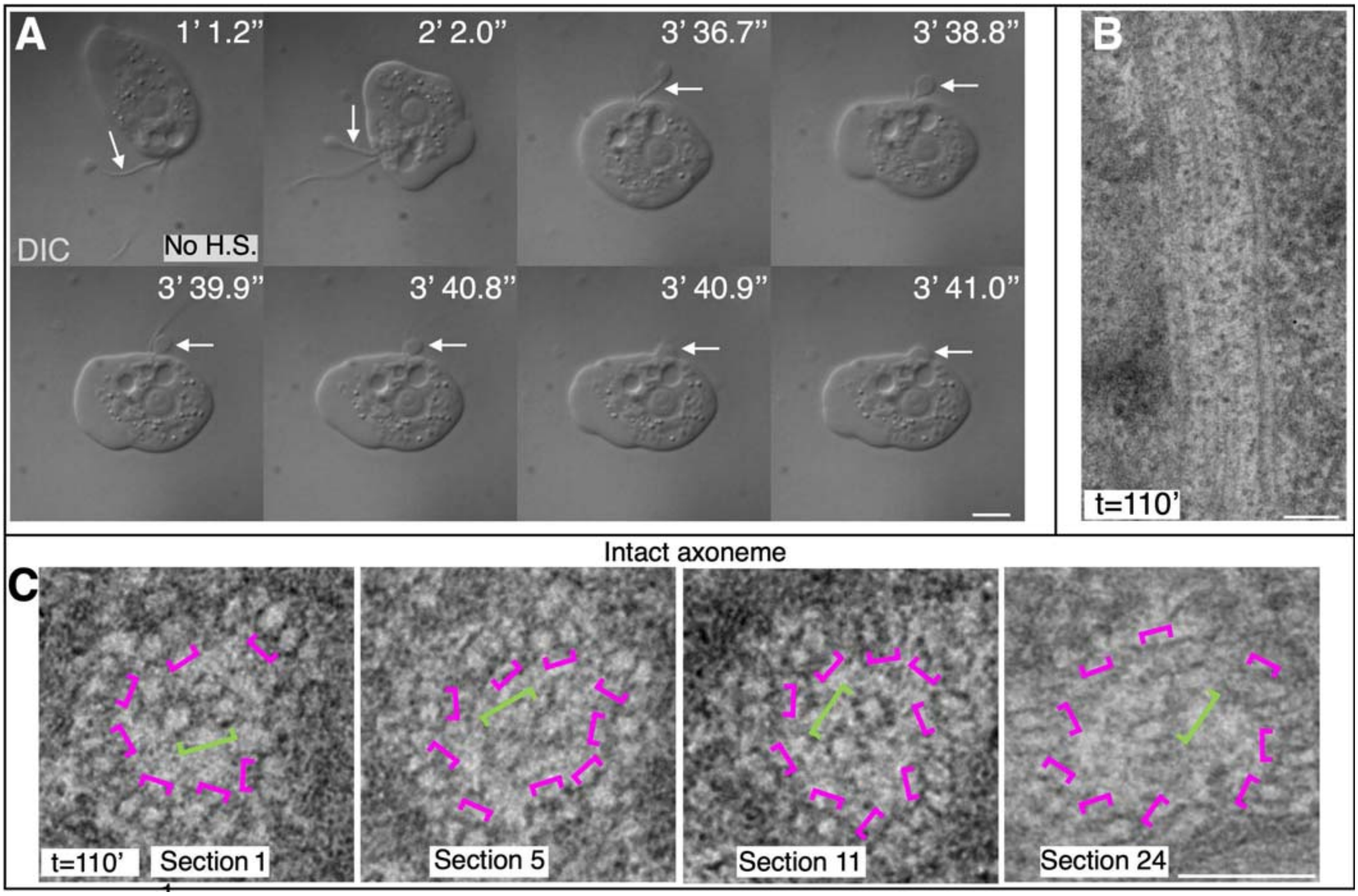

Figure EV2. Axoneme internalization mechanisms.

(**A**) Still images of DIC movie of flagellated cell (~$t = 100$ min after transformation onset). Reversal was induced by pressure of the coverslip. Time in min and sec since the beginning of the movie. Arrow: retracting flagellum. Note that a bulge is visible at the end of the retracting flagellum throughout the recording. The second flagellum is present throughout the recording, but out of focus until the first one is completely retracted. In the remainder of the movie, the second flagellum is internalized later by folding back on the cell body and subsequent membrane fusion. Scale bar is 5 µm. (**B**) Single section EM from a serial section series of an internalized axoneme in side view, highlighting the lack of membranes surrounding the microtubule wall at $t = 100$ min after transformation onset. Heat shocked at $t = 90$ min. Scale bar is 100 nm. (**C**) Top views of select 50 nm EM sections from 24 consecutive serial section series from an intact internalized axoneme, from the region shown in Fig. 3E. Microtubule doublets are indicated by magenta brackets, the pair of central microtubules by a green bracket. Note that microtubules are present in all sections (i.e., spanning >1 µm in total). Scale bar is 100 nm.

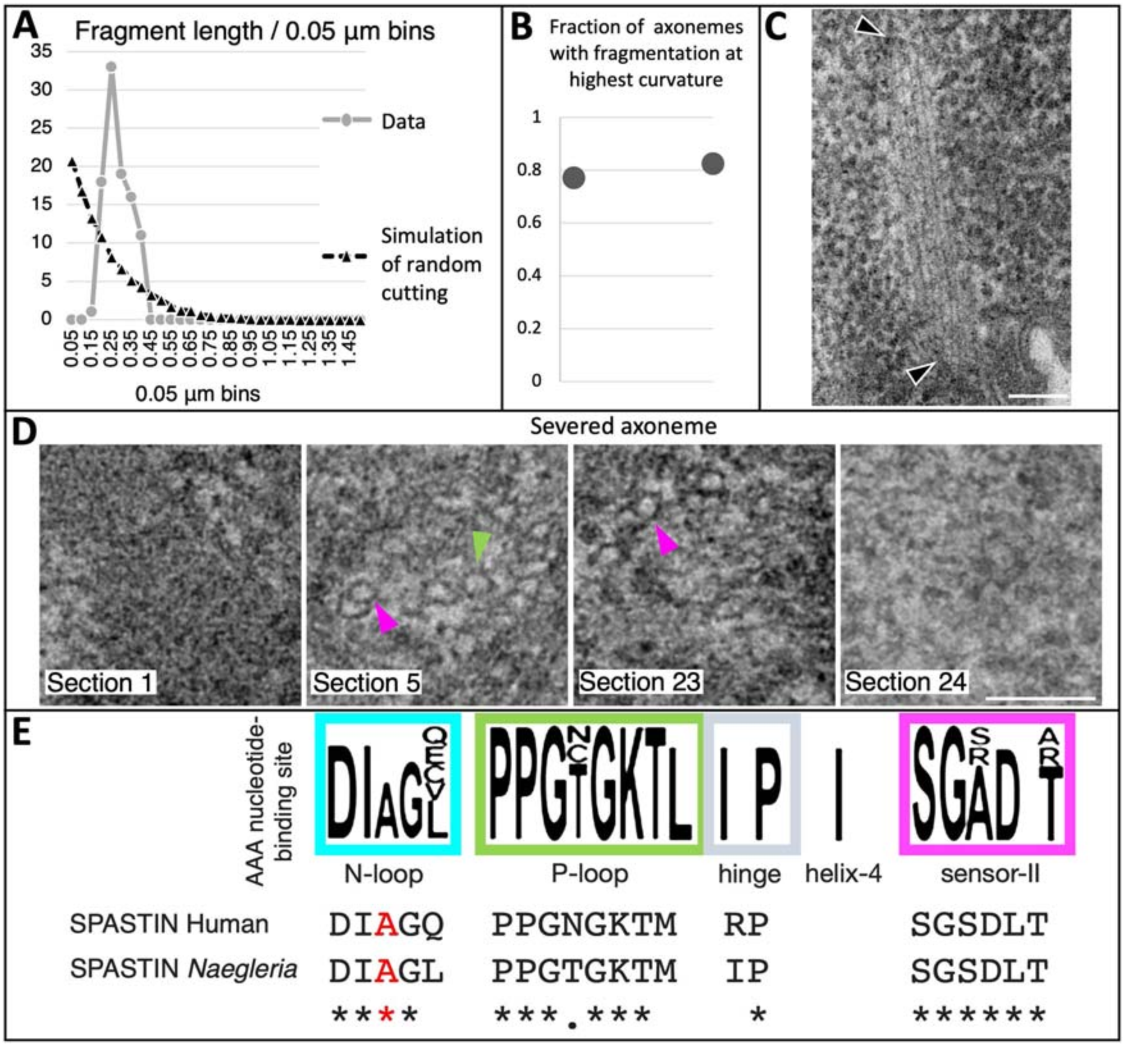

Figure EV3. Axonemes are severed by Spastin in regions of highest curvature.

(**A**) Distribution of fragment length determined in the data (gray discs, binned into 0.05 µm bins; $N = 98$ fragments) or simulated considering random cutting (black triangles). (**B**) Fraction of axonemes in which the severed region coincides with the region of highest curvature. Two experimental replicates ($N = 74$ and 52 axonemes, respectively). (**C**) Single section EM from a serial section series of an axonemal fragment in side view. Note that the fragment is ~500-nm long. Black arrowheads indicate the two ends of the fragment. Scale bar is 100 nm. (**D**) Top views of select 50 nm EM sections from 24 consecutive serial section series from an axonemal fragment, from the region shown in Fig. 3E. Microtubule doublets are indicated by magenta arrowheads, central microtubules by green arrowhead. Note that microtubules are present only between sections 5 and 23 (covering ~900 nm), indicative of this element being larger than the average axonemal fragment. Scale bar is 100 nm. (**E**) Sequence logo diagram for residues in the nucleotide-binding site of AAA domains that was used to design a specific inhibitor for Spastin (top, from Cupido et al, 2019), as well as alignment of the relevant regions in human and *Naegleria* Spastin (bottom). Spastin Uniprot accession number: D2VS83 (Fritz-Laylin et al, 2010). Note that the N-terminal part of the protein was not correctly annotated and is thus missing in the accession number entry; Uniprot was contacted to correct this. Note also that mutating the "A" in "DIAGO" (highlighted in red) abolishes Spatozoline action on human Spastin (Cupido et al, 2019).

 © The Author(s)

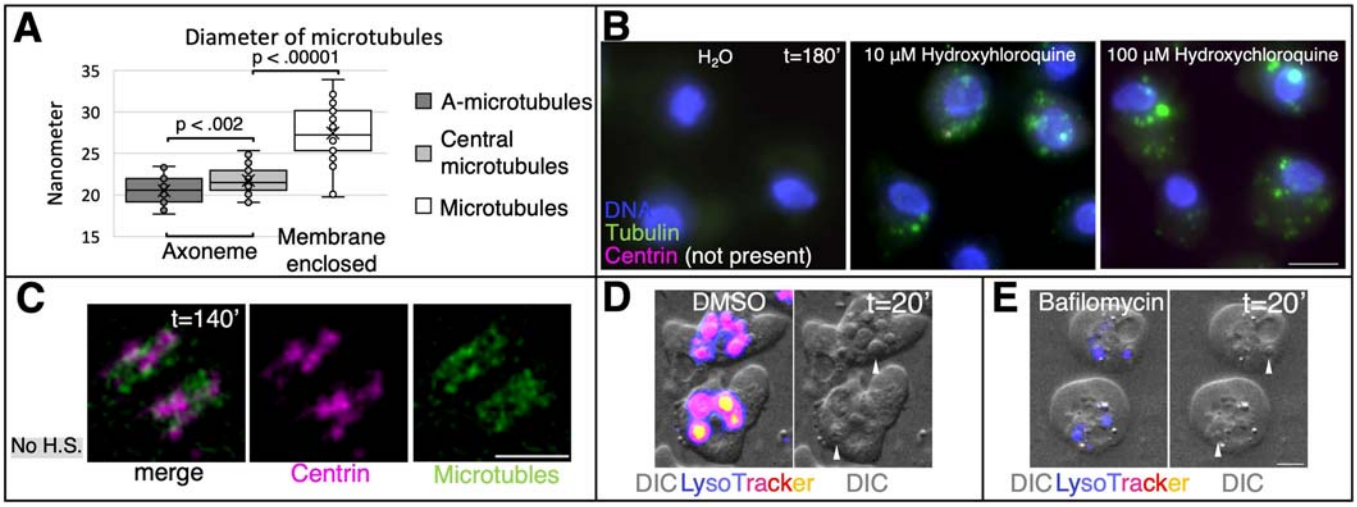

Figure EV4. Microtubule diameter in membrane enclosed vesicles and axoneme fragments, as well as evidence that LysoTracker labels lysosomes in *Naegleria* and that tubulin positive elements are autophagosome.

(**A**) Quantification of microtubule diameters from top views in indicated compartments. Diameters were determined by measuring the perimeter of a fitted circle in individual sections. A-microtubules: $N = 35$ measurements in different sections of 18 microtubules from two axonemes in cross section. Central microtubules: $N = 36$ measurements in different sections of 4 microtubules from two axonemes in cross section. Membrane-enclosed microtubules: $N = 29$ measurements from independent membrane-enclosed elements. Boxes represent the interquartile range (IQR), containing the middle 50% of the data. Line represents the median, the cross the mean. Whiskers extend from the first (Q1) and third quartile (Q3) down and up to the smallest and largest value within 1.5 times the IQR below Q1 and above Q3. All data points including outliers are presented. A one-way ANOVA test was performed with the resulting p value of 1E−15, followed by a Tukey-Kramer post hoc test with the resulting studentized range distributions of T1:T2 Q = 3.09 ($p = 0.07899$), T1:T3 Q = 17.54 ($p = 0.00000$), and T2:T3 Q = 14.45 ($p = 0.00000$). Note that the diameter of the central pair of microtubules in the axoneme is larger than that of the A-microtubule in the peripheral microtubule doublets. (**B**) Cells heat shocked for 20 min at $t = 95$ min and incubated with 0.1% DMSO (left), 10 μM (middle), or 100 μM Hydroxychloroquine (right); cells were fixed at $t = 180$ min and immunostained for α/β tubulin (green) and Centrin (magenta); DNA is visible in blue. Scale bar is 5 μm. (**C**) Single plane STED images of centrioles from cells fixed at 105 min, immunostained for Centrin (magenta). Tubulin is visualized with the cabazitaxel-derived microtubule probe Sir-Tubulin (labeled with Rhodamine, pseudo-colored in green). Scale bar is 500 nm. (**D**, **E**) Representative single plane DIC and fluorescence images of live cells treated with DMSO or Bafilomycin at $t = 0$ min, imaged at $t = 20$ min after onset of transformation. LysoTracker was added 10 min before imaging. Note that LysoTracker signals can be observed in vesicles. LysoTracker signals are displayed in the ImageJ LUT "Fire", which displays higher gray values in red-yellow and lower gray values in blue-purple color code. White arrowheads indicate examplary vesicles visible by DIC. Scale bar in (**E**) (for **D** and **E**) is 5 μm.

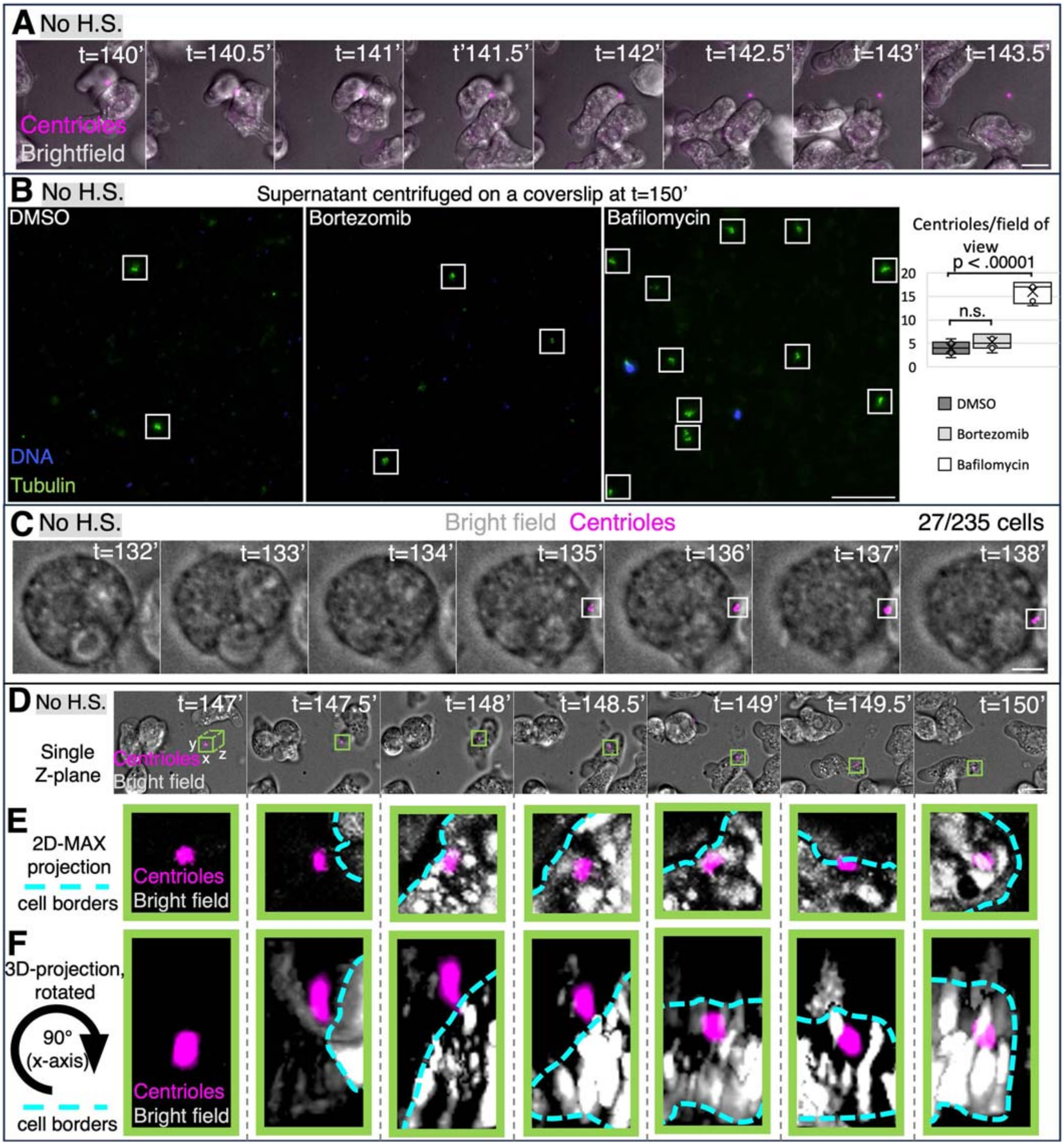
A No H.S.
t=140'
t=140.5'
t=141'
t'141.5'
t=142'
t=142.5'
t=143'
t=143.5'
Centrioles
Brightfield
B No H.S.
Supernatant centrifuged on a coverslip at t=150'
DMSO
Bortezomib
Bafilomycin
DNA
Tubulin
Centrioles/field of view
p < .00001
n.s.
20
15
10
5
0
DMSO
Bortezomib
Bafilomycin
C No H.S.
Bright field Centrioles
27/235 cells
t=132'
t=133'
t=134'
t=135'
t=136'
t=137'
t=138'
D No H.S.
Single Z-plane
t=147'
t=147.5'
t=148'
t=148.5'
t=149'
t=149.5'
t=150'
Centrioles
Bright field
E
2D-MAX projection
cell borders
Centrioles
Bright field
F
3D-projection, rotated
90° (x-axis)
cell borders
Centrioles
Bright field

 © The Author(s)

Figure EV5. Centrioles can be shed in the external environment and taken up by other cells.

(**A**) All frames of the movie displayed in Fig. 5H. Time is shown in min since transformation onset. Scale bar is 10 µm. (**B**) The supernatant of a culture at $t = 150$ min after transformation onset, following a 20 min heat shock at $t = 90$ min, was centrifuged onto a coverslip at 10,000 rcf for 5 min after removal of cells by centrifugation for 2 min at 750 rcf. This was followed by immunostaining for α/β tubulin (green); DNA is visible in blue. $N = 6$ (0.1% DMSO), 7 (25 µM Bortezomib) and 5 (10 µM Bafilomycin A1) fields of view from one representative experiment. (right) Corresponding quantification. Boxes represent the interquartile range (IQR), containing the middle 50% of the data. Line represents the median, the cross the mean. Whiskers extend from the first (Q1) and third quartile (Q3) down and up to the smallest and largest value within 1.5 times the IQR below Q1 and above Q3. All data points including outliers are presented. A one-way ANOVA test was performed with the resulting p value of 128E−8, followed by a Tukey-Kramer post hoc test with the resulting studentized range distributions of T1:T2 Q = 1.79 ($p = 0.43401$), T1:T3 Q = 16.73 ($p = 0.00000$) and T2:T3 Q = 14.94 ($p = 0.00000$). Note that from the ~3-fold increase in centriole shedding estimated from the number of centrioles recovered on coverslips upon Bafilomycin A1 addition, one would expect ~30% of cells lacking centrioles (i.e., 3 times ~10% of cells shedding centrioles). However, this was the case in only ~10% of Bafilomycin A1-treated cells (see Fig. 6A). One possibility to explain this discrepancy is that centrioles shed in control conditions are more fragile than those released upon Bafilomycin A1 treatment, leading to an underestimation when counting those left on the coverslip. Scale bar is 10 µm. (**C**) Stills of partially 2D-projected 3D stacks of widefield microscopy time-lapse at indicated time points after transformation onset. Amoeboid cells were immobilized with 10 µM Latrunculin B at the beginning of the recording, when LysoTracker and the cabazitaxel-derived microtubule probe SPY650 were added. Note that in this particular case SPY650 was not added at the onset of transformation, which leads to labeling of ~50% of centrioles, but only at the beginning of the movie ($t = 130'$). Thus, centrioles are not detected whilst still in the cell, presumably due to the time it takes SPY650 needs to penetrate the cell and bind centrioles, but become visible upon their shedding (at $t = 135'$). Shedding was observed in 27/235 cells analyzed. Scale bar is 5 µm. (**D**) Single planes of all frames of the Z-stack movie displayed in Fig. 5I. Green box indicates regions highlighted in (**E**) and (**F**). Time in min since transformation onset. Scale bar is 10 µm. (**E**) Z-max projection of the region boxed in D containing the centriole. (**F**) 3D projection of the region boxed in (**D**), rotated by 90° to enable viewing the cell "from the side". Note that until $t = 148.5$ min, centrioles are outside the cell (cell borders, which were determined by visual inspection in 3D, are highlighted by a cyan dashed line in 3D projections at $t = 148$ and 148.5 min; this is visible only in the 3D projection). By contrast, starting at $t = 149$ min, the centrioles are inside the new host.

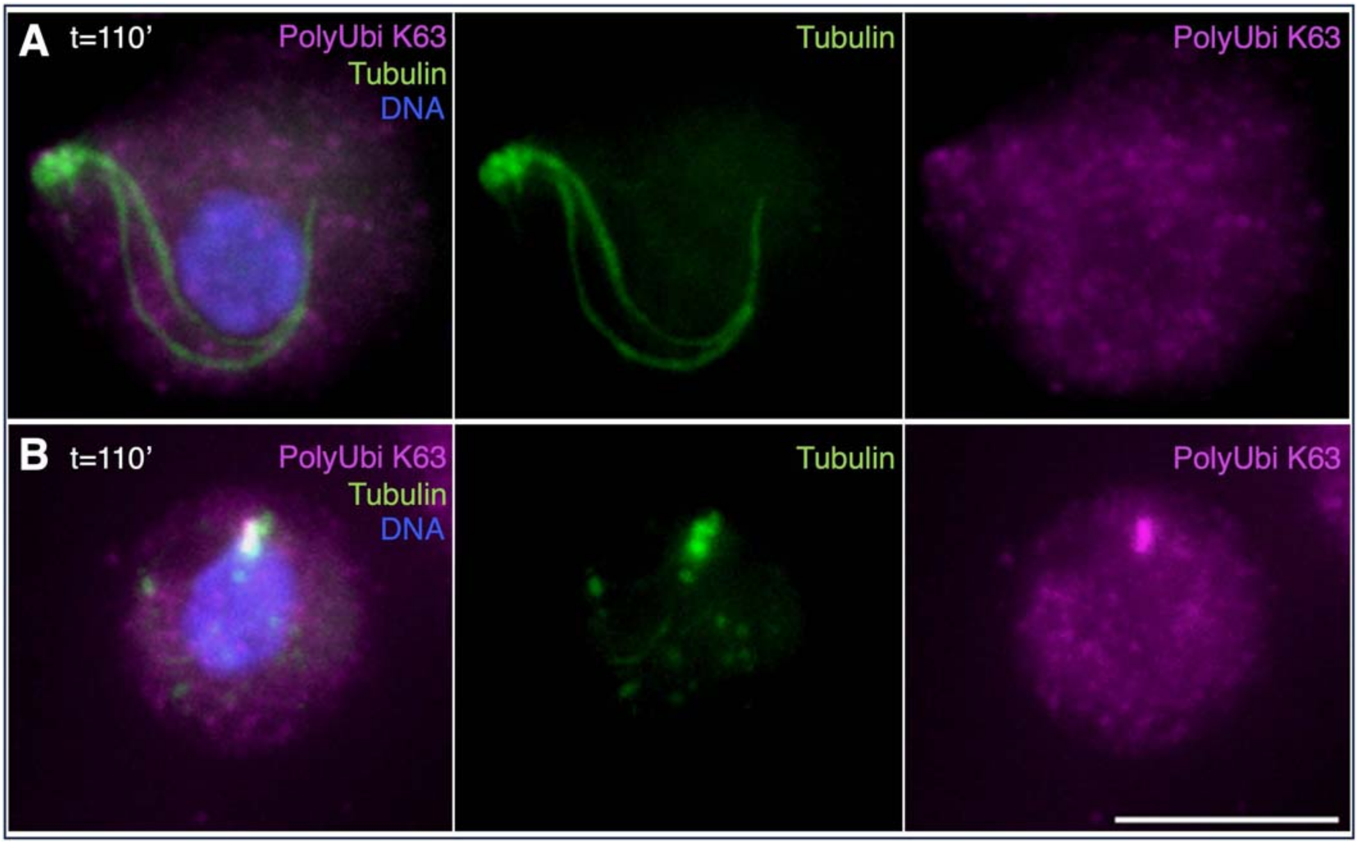

Figure EV6. Centrioles, but not flagella or flagellar fragments, harbor K63-linked poly-ubiquitination.

(**A**, **B**) Cells heat shocked at $t = 95$ min and fixed at $t = 110$ min were immunostained with antibodies against α/β tubulin (green), as well as K63-linked poly-ubiquitination (magenta); DNA is visible in blue. The majority of cells at this time point resemble that shown in (**A**), whereas a minority of them have already undergone axonemal fragmentation (**B**). Note that K63-linked poly-ubiquitinated antibodies do not mark the axoneme (**A**) or tubulin positive spots (**B**), but start to highlight centrioles at the onset of elimination. Scale bar is 5 µm.

 © The Author(s)
